# Trends and projections of PM_2.5_-attributable disease burden in China: a GBD 2021-based analysis

**DOI:** 10.3389/fpubh.2026.1684344

**Published:** 2026-01-15

**Authors:** Tao Fang, Yang Xu, Na Shen, JinYang Liu, Yanbo Di, Shike Hou

**Affiliations:** 1 Institute of Disaster and Emergency Medicine, Tianjin University, Tianjin, China; 2 Central Laboratory, Tianjin 4th Center Hospital, Tianjin, China; 3 Department of Medicine, Tianjin Medical College, Tianjin, China; 4 Clinical Laboratory, Tianjin 4th Center Hospital, Tianjin, China

**Keywords:** age-period-cohort analysis, disease burden, fine particulate matter, prediction, temporal trend

## Abstract

**Background:**

Fine particulate matter (PM_2.5_) remains a major environmental health risk in China. This study aimed to comprehensively characterize the long-term trends and epidemiological patterns of PM_2.5_-attributable disease burden across the country.

**Methods:**

Using data from the Global Burden of Disease (GBD) 2021, we performed a longitudinal analysis of PM_2.5_ disease burden from 1990 to 2021. Joinpoint regression was employed to calculate the average annual percentage change (AAPC) in age-standardized rates. The age-period-cohort model was employed to stratify the impact of age, periods, and birth cohorts on disease burden. Finally, the Bayesian age-period-cohort (BAPC) analysis was used to project trends for the period 2022–2036.

**Results:**

In 2021, PM_2.5_ exposure was responsible for an estimated 2.27 million deaths and 46.68 million disability-adjusted life years (DALYs) in China. Compared to 1990, the age-standardized mortality rate declined to 125 per 100,000 (AAPC = −3.62, net drift = −4.19), while the DALY rate fell to 2,437 per 100,000 (AAPC = −3.93, net drift = −3.87). The disease burden from ambient PM_2.5_ increased substantially and became the dominant source by 2021, whereas that from household PM_2.5_ decreased markedly. Overall, males experienced a higher disease burden than females. Notably, the 30–34 age group showed rising trends in ambient PM_2.5_ burden, while children under five and older adults remained the most vulnerable groups. Bayesian projections indicate that total and ambient PM_2.5_ burdens may continue to decline through 2036, although household PM_2.5_ could rebound slightly.

**Conclusion:**

Although substantial improvements in air quality have been achieved, pronounced disparities persist between PM_2.5_ sources and population subgroups. Strengthening targeted and equitable public health interventions remains essential to sustain progress and reduce the unequal health risks associated with PM_2.5_ exposure in China.

## 1 Introduction

Particulate matter with an aerodynamic diameter of less than 2.5 μm (PM_2.5_) has emerged as a major environmental risk factor, contributing substantially to global morbidity and mortality (1–4). In 2021, more than 7 million deaths worldwide were attributed to particulate matter pollution (PMP, total PM_2.5_), making it the leading environmental and occupational risk factor globally (5). Numerous epidemiological studies have demonstrated that both short-term and long-term exposure to PM_2.5_ increases the risk of a wide range of adverse health outcomes, even at relatively low concentrations (6, 7). The health effects of PM_2.5_ are mediated through multiple molecular mechanisms, including oxidative stress, cytokine release, DNA damage, alterations in gene expression, immunotoxicity, inflammation, and apoptosis. These biological processes collectively trigger or exacerbate respiratory, cardiovascular, metabolic, and other systemic diseases (8–10).

China, with its vast geographic, climatic, and socioeconomic diversity, faces the dual challenges of ambient fine particulate pollution (APMP, ambient PM_2.5_) and household air pollution from solid fuels (HAP-SF, household PM_2.5_) (11, 12). Rapid urbanization and industrial expansion have led to a substantial increase in ambient PM₂.₅ concentrations, particularly in urban and industrial regions (13). Meanwhile, the implementation of large-scale clean energy programs has markedly reduced indoor air pollution exposure, especially in rural areas (14). These evolving trends in ambient and household PM_2.5_ underscore the need for source-specific and demographically targeted assessments of disease burden. Additionally, understanding how PM_2.5_-attributable disease burden varies across age, period, and cohort will be beneficial for tailoring public health responses. We used the age-period-cohort analysis, which offers a nuanced framework to disentangle the effects of biological aging (age), time-specific societal or environmental changes (period), and shared generational exposures (cohort), providing more comprehensive insight than traditional trend analyses (15).

In this study, we performed a comprehensive longitudinal analysis of disease burden attributable to total, ambient and household PM_2.5_ exposure in China from 1990 to 2021. Based on Global Burden of Disease (GBD) 2021 data, we utilized joinpoint regression, age-period-cohort modeling, and Bayesian age-period-cohort (BAPC) analysis to analyze historical trends and predict disease burden up to 2036. This descriptive analysis builds upon previous findings, offering updated insights into the long-term trends and projections, and aims to elucidate the evolving dynamics of health impacts attributable to PM_2.5_, informing targeted mitigation efforts in China and other rapidly developing nations.

## 2 Methods

### 2.1 Overview

Within the GBD 2021 framework, total PM_2.5_ is classified as a level 3 risk factor, encompassing two level 4 risk factors: ambient PM_2.5_ and household PM_2.5_. Annual estimates of mortality, disability-adjusted life years (DALYs), and age-standardized rates (ASRs) attributable to PM_2.5_ in China from 1990 to 2021 were obtained from the Global Health Data Exchange GBD Results Tool. All estimates were accompanied by 95% uncertainty intervals (UIs), defined as the range between the 2.5th and 97.5th percentiles, and were stratified by sex (male, female, and both sexes combined). Population data were stratified into 20 distinct groups, each spanning 5 years, ranging from <5 years to ≥95 years. In GBD 2021, solid fuels are defined as coal and charcoal, wood, crop residues, and dung (16). Details of the estimation framework and statistical methods used to assess the health burden attributable to different sources of PM_2.5_ exposure, including ambient and household, have been described in previous publications (5).

This study was exempt from institutional ethics review because all data used were obtained from the publicly accessible GBD 2021 database.

### 2.2 Jointpoint regression analysis

Trends in age-standardized mortality (ASMR) and DALY rates (ASDR) from 1990 to 2021 were analyzed using the Joinpoint Regression Program (v5.4.0) to estimate annual percentage change (APC) and average annual percentage change (AAPC) with 95% confidence intervals (CIs). Trends were considered increasing or decreasing if the 95% CI was entirely above or below 0, respectively, and stable if it included 0. If the 95% CI included 0, the trend was considered stable. A *p*-value < 0.05 was considered statistically significant (17, 18).

### 2.3 Age-period-cohort analysis

This study employed the age-period-cohort model framework to analyze the underlying trends in mortality and DALYs by age, period, and birth cohort (19). To address the linear dependency among age, period, and cohort (birth cohort = period - age), we employed the age-period-cohort web tool, which resolves the issue by generating estimable age-period-cohort parameters and functions without imposing arbitrary constraints on model parameters (15, 20). A typical age-period-cohort model requires five-year grouped intervals for both age and calendar period. Age data were classified into 20 five-year groups, starting with age_ < 5, age_5–9, age_10–14, and continuing up to age_ ≥ 95. The study period was divided into six five-year intervals; for example, “period_1992” denotes the period from 1992 to 1996, while “period_2017” denotes the period from 2017 to 2021. Lastly, 25 overlapping five-year birth cohorts were constructed, beginning with individuals born between 1897 and 1901 (cohort_1897) and ending with those born between 2017 and 2021 (cohort_2017). The age-period-cohort indicators employed in this study include net drift, local drift, longitudinal age curves (age effect), period relative risk (period effect), and cohort relative risk (cohort effect). The net drift is defined as the overall annual percentage change in disease burden across all ages, while the local drift is defined as the annual percentage change within each age group, reflecting age-specific temporal trends. The relative risk is calculated as the ratio of age-specific rates in each period (or cohort) relative to a reference period (or cohort). The selection of the reference period (or cohort) is arbitrary and does not influence the interpretation of the results. The Wald χ^2^ test was employed for statistical analysis, with a *p*-value < 0.05 as statistically significant (21, 22).

### 2.4 Bayesian age-period-cohort analysis

The BAPC analysis was implemented using the BAPC package in R (version 4.4.2), which is based on the integrated nested Laplace approximations (INLA) framework, to project the PM_2.5_-attributable ASMR and ASDR in China from 2022 to 2036. Within the Bayesian framework, age, period, and cohort effects were modeled with a second-order random walk prior to ensure smooth temporal trends. INLA was used to directly approximate the posterior marginal distributions without the need for Markov Chain Monte Carlo sampling, thus avoiding potential issues related to mixing and convergence (23–25). The outstanding predictive performance of the BAPC model has been validated previously, for it can facilitate efficient estimation of latent effects and provides valuable insights into the dynamic interactions among age, period, and cohort factors (26). Standard population weights and population forecasts were obtained from the GBD database, and all analyses and visualizations were conducted in R (17, 27).

## 3 Result

### 3.1 Descriptive analysis

In 2021, total PM_2.5_ exposure was responsible for an estimated 2.27 million deaths and 46.68 million DALYs in China. Among these, ambient PM_2.5_ contributed the most, accounting for 1.86 million deaths and 37.81 million DALYs, while household PM_2.5_ contributed with 0.42 million deaths and 8.86 million DALYs. Compared to 1990, the disease burden from ambient PM_2.5_ showed a sharp increase, with deaths rising by over 200% and DALYs increasing by nearly 200%. In contrast, the burden from household PM_2.5_ declined significantly, with deaths falling by 77% and DALYs decreasing by 83%. The detailed disease burdens for total, ambient and household PM_2.5_ are illustrated in Table 1 and Supplementary Table S1. The disease burden in males was higher than in females across all measures.

**Table 1:** Time trends of PM_2.5_-attributable mortality in China, 1990–2021, stratified by sex.

Characteristics	1990		2021		1990–2021	Net drift^†^
	All-ages cases (million) *n* (95% UI)	ASMR per 100,000 *n* (95% UI)	All-ages cases (million) *n* (95% UI)	ASMR per 100,000 *n* (95% UI)	AAPC in ASMR (95% CI)	% per year
PMP						
Both	2.24 (1.92–2.57)	367.58 (317.05–421.27)	2.27 (1.77–2.89)	125.13 (97.59–157.87)	−3.62^*^ (−4.10–−3.14)	−4.19^*^ (−4.40–−3.97)
Female	1.06 (0.88–1.25)	320.45 (264.24–378.43)	0.97 (0.72–1.24)	94.81 (70.02–121.69)	−4.06^*^ (−4.55–−3.57)	−5.20^*^ (−5.44–−4.96)
Male	1.18 (0.97–1.41)	440.07 (370.00–513.53)	1.31 (0.99–1.73)	169.65 (131.05–221.14)	−3.11^*^ (−3.51–−2.71)	−3.60^*^ (−3.88–−3.32)
APMP						
Both	0.45 (0.22–0.79)	74.83 (35.44–131.25)	1.86 (1.30–2.29)	102.34 (71.92–126.30)	0.92^*^ (0.19–1.65)	1.20^*^ (0.94–1.46)
Female	0.18 (0.08–0.32)	55.19 (25.25–98.95)	0.76 (0.50–1.00)	74.96 (49.08–98.01)	0.92^*^ (0.36–1.47)	0.73^*^ (0.42–1.03)
Male	0.27 (0.13–0.49)	104.89 (49.02–184.01)	1.09 (0.77–1.40)	142.53 (101.51–178.93)	0.97^*^ (0.62–1.32)	1.40^*^ (1.10–1.71)
HAP-SF						
Both	1.79 (1.40–2.14)	292.73 (231.22–351.53)	0.42 (0.06–1.35)	22.76 (3.52–73.91)	−8.02^*^ (−8.82–−7.21)	−9.56^*^ (−9.79–− 9.34)
Female	0.88 (0.70–1.08)	265.24 (209.01–324.02)	0.20 (0.03–0.63)	19.82 (3.23–61.09)	−8.13^*^ (−8.98–−7.27)	−10.02^*^ (−10.28–−9.77)
Male	0.91 (0.68–1.13)	335.18 (249.14–411.20)	0.21 (0.03–0.75)	27.08 (3.65–96.74)	−7.92^*^ (−8.94–−6.89)	−9.26^*^ (−9.54–−8.99)

ASMR, age-standardized mortality rate; UI, uncertainty interval; AAPC, average annual percentage change; CI, confidential interval; *, *p* < 0.05.

† Net drifts are estimates derived from the age-period-cohort model and denotes overall annual percent change in mortality, with values expressed as percent change per year (% per year).

**Figure 1:**
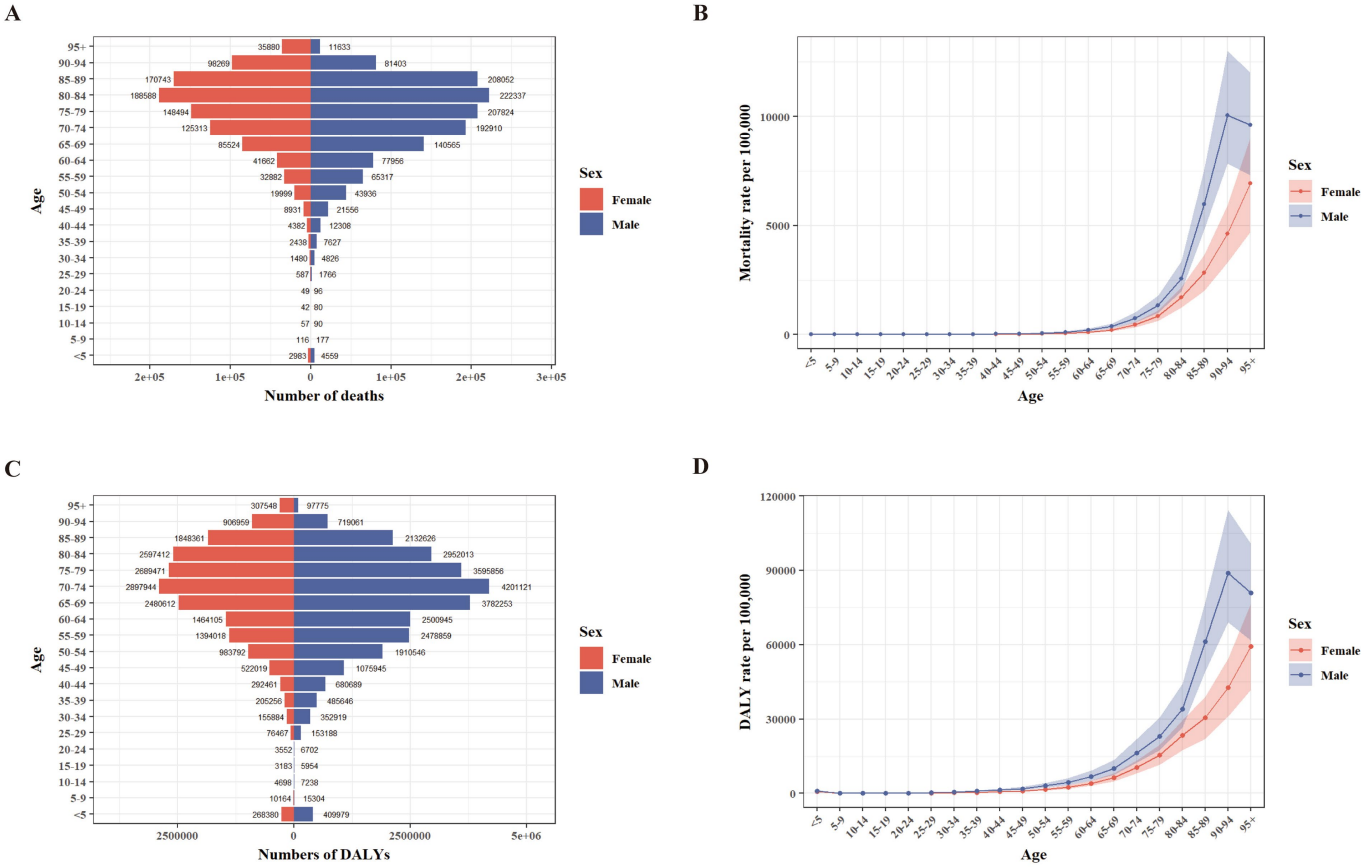
Age-stratified counts and rates of deaths and DALYs from total PM_2.5_ in China, 2021, by sex **(A–D)**. **(A)** Death counts. **(B)** Death rates. **(C)** DALY counts. **(D)** DALY rates. DALY, disability-adjusted life year.

The mortality and DALY numbers, as well as age-specific rates of total, ambient, and household PM_2.5_, across different age groups in 2021 are presented in Figure 1 and Supplementary Figures S1, S2. Mortality was highest in individuals aged 80–84 years for both sexes, whereas DALYs peaked in the 70–74 age group. Age-specific mortality and DALY rates reached their maximum in males aged 90–94 years and females aged 95 + years. In most age groups, males exhibited higher mortality and DALY numbers and rates than females, with only a few exceptions.

### 3.2 Temporal trend in the disease burden attributable to PM_2.5_

**Figure 2:**
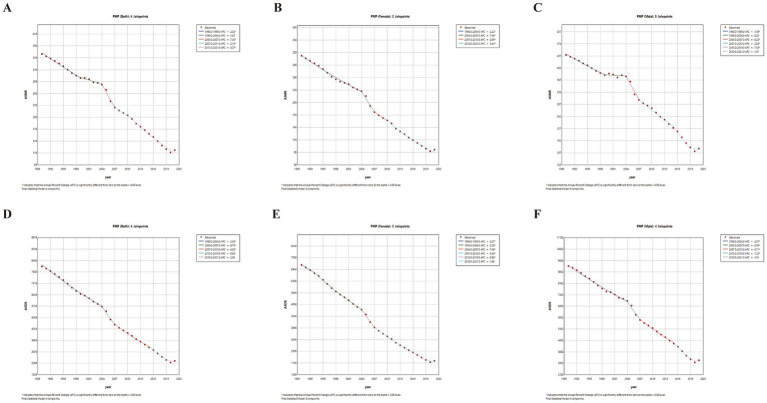
Trends in total PM_2.5_-attributable disease burden from 1990 to 2021. **(A–C)** ASMR for both sexes, females, and males. **(D–F)** ASDR for both sexes, females, and males. ASMR, age-standardized mortality rate; ASDR, age-standardized DALY rate; APC, annual percentage change; ^*^, *p* < 0.05.

We conducted joinpoint regression analyses to evaluate temporal trends in ASRs attributable to total, ambient, and household PM_2.5_ (Table 1; Supplementary Table S1; Figure 2; Supplementary Figures S3, S4). For total PM_2.5_, both the ASMR (APCC: −3.62 [95% CI: −4.10 to −3.14]) and ASDR (AAPC: −3.93 [−4.22 to −3.65]) showed significant declines over the study period, with the most significant decreases for ASMR occurring from 2004 to 2007 for both sexes (APC: −7.30 [−10.91 to −3.55]), and from 2015 to 2019 for ASDR (APC: −6.85 [−7.99 to −5.69]). In contrast, the disease burden from ambient PM_2.5_ showed fluctuations. The AAPCs for ambient PM_2.5_-attributable ASMR and ASDR were 0.92 (0.19 to 1.64) and 0.59 (−0.06 to 1.24), respectively, reflecting an overall upward trend during 1990–2021. Both indicators rose steadily from 1990, reaching their highest values in 2014, followed by a sustained decline through to 2021. For household PM_2.5_, there was a consistent and significant downward trend in both ASMR (AAPC: −8.02 [−8.82 to −7.21]) and ASDR (AAPC: −8.28 (−8.93 to −7.62)) from 1990 to 2021. 1.

### 3.3 Net drift and local drift of PM_2.5_-attributable mortality and DALYs across age groups

**Figure 3:**
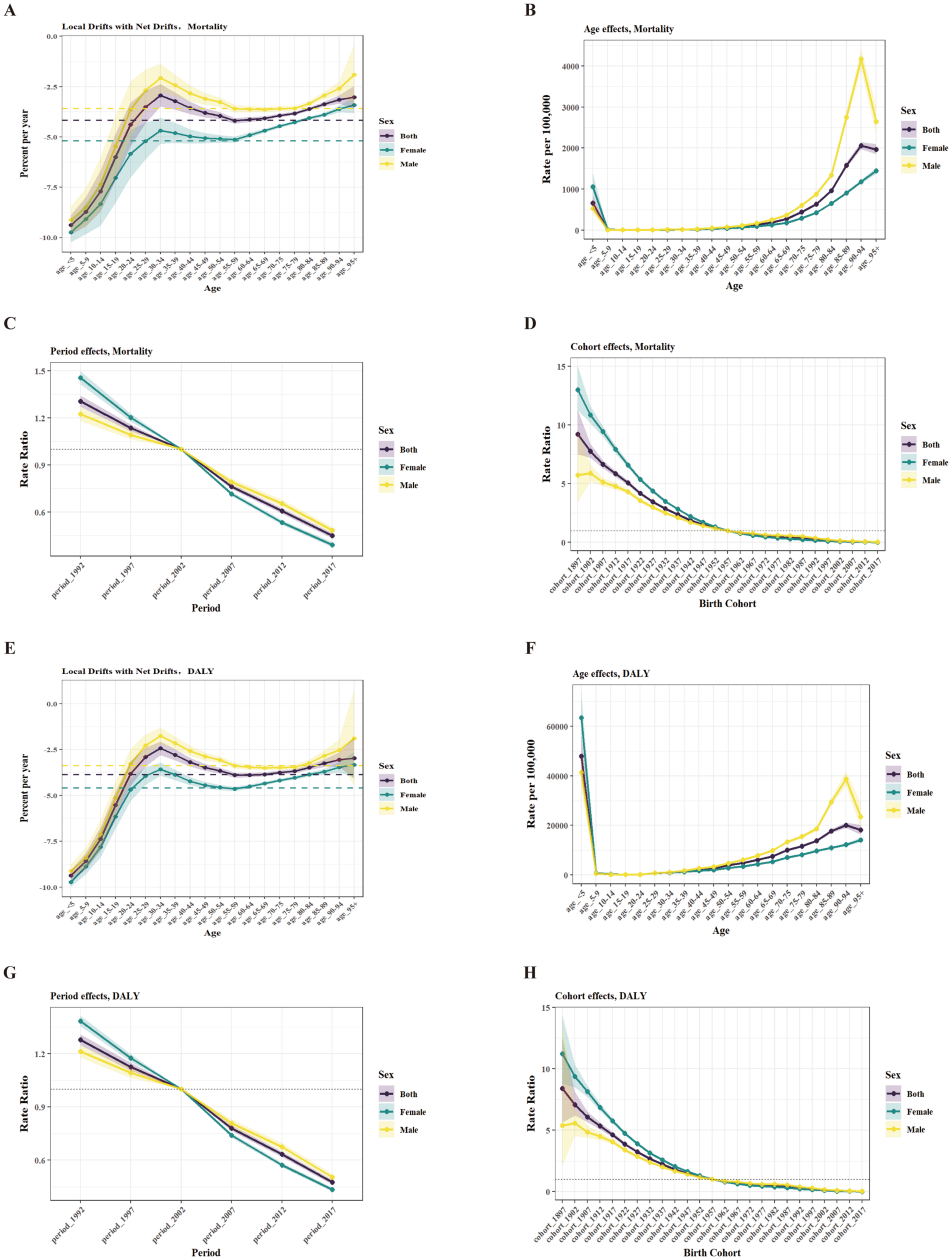
Age-period-cohort effects on total PM_2.5_-attributable disease burden. **(A)** Local drift in mortality (95% CI). The dashed horizontal lines indicate the net drift. **(B)** Mortality longitudinal age curves (95% CI). **(C)** Mortality period rate ratios vs. reference (95% CI). **(D)** Mortality cohort rate ratios vs. reference (95% CI). **(E)** Local drift in DALY (95% CI). The dashed horizontal line indicates the net drift. **(F)** DALY longitudinal age curves (95% CI). **(G)** DALY period rate ratios vs. reference (95% CI). **(H)** DALY cohort rate ratios vs. reference (95% CI). DALY, disability-adjusted life year; CI, confidence interval.

Figure 3, Supplementary Figures S5–S6, Table 1, and Supplementary Tables S1–S4 illustrate the net drift and local drift values for mortality and DALY attributable to total, ambient, and household PM_2.5_ in China. Over the study period, the disease burden attributable to total PM_2.5_ and household PM_2.5_ showed a significant downward trend, whereas the burden attributable to ambient PM_2.5_ increased. The net drifts in mortality attributable to both sexes were −4.19 (95% CI: −4.40 to −3.97) for total PM_2.5_, 1.20 (0.94 to 1.46) for ambient PM_2.5_, and −9.56 (−9.79 to −9.34) for household PM_2.5_, respectively. Corresponding net drift values for DALYs were −3.87 (−4.01 to −3.72), 1.52 (1.37 to 1.67), and −9.22 (−9.37 to −9.08), respectively.

The local drift curves for mortality and DALY exhibited similar overall patterns across all sex groups and PM_2.5_ sources. Specifically, local drift values were lowest in the age_ < 5 group, gradually increased with age, and generally peaked around 30–34 age group, with minor variations across subgroups. Thereafter, the values exhibit a U-shaped pattern, reaching a second high point in the age_ 95 + group.

### 3.4 The effect of age, period and cohort on PM_2.5_ mortality and DALY rates

Figure 3, Supplementary Figures S5, S6, and Supplementary Tables S5–S13 present the estimated age, period, and cohort effects on mortality and DALYs from total, ambient, and household PM_2.5_ in China. Longitudinal age curves showed that total PM_2.5_ mortality rates peaked in the age_90–94 for both sexes and males, and in the age_95 + for females, while DALY rates were highest in age_ < 5 group across all sex groups. For ambient PM_2.5_, mortality and DALY rates peaked in age_95 + group for both sexes and females, and in the age_90–94 group for males. Household PM_2.5_ mortality and DALY rates peaked in age_ < 5 group for all sexes.

Period effects showed a consistent decline in mortality and DALY risks for total and household PM_2.5_ from 1992 to 2021, with the highest risks in period 1992–1996 and the lowest in period 2017–2021. For ambient PM_2.5_, the risks were maximized during the period 2012–2016 and minimized during the period 1992–1996, regardless of sex.

Over 25 birth cohorts (1987–2021), cohort risks for total and household PM_2.5_ continued to decrease, while those for ambient PM_2.5_ initially increased and then declined. Ambient PM_2.5_ mortality risks peaked in the 1987–1991 cohort for both sexes and females, while males saw the peak in 1992–1996. DALY risks for ambient PM_2.5_ peaked in the 1992–1996 cohort for both sexes and males, while females saw the peak in 1987–1991 cohort.

### 3.5 Projection for disease burdens attributable to PM_2.5_ from 2022 to 2036

**Figure 4:**
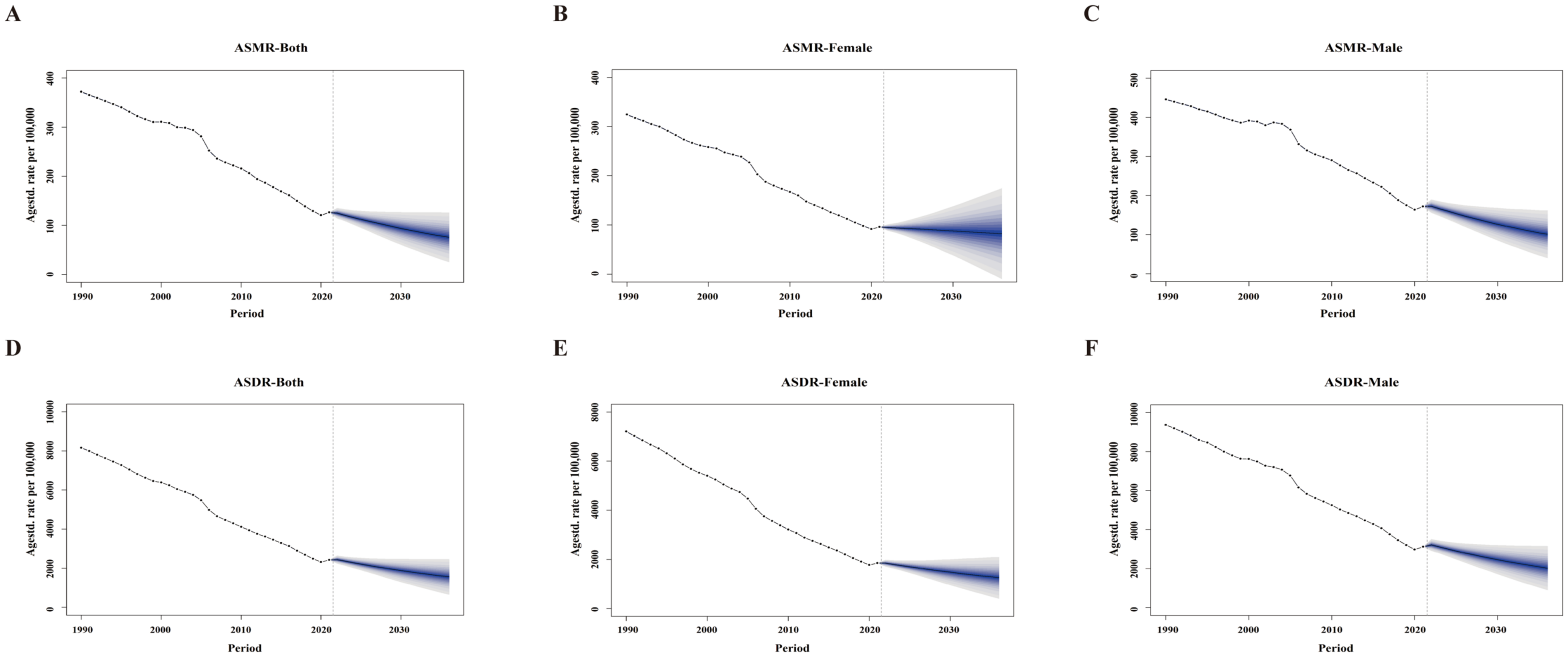
Projected total PM_2.5_-attributable disease burden from 1990 to 2021. **(A–C)** ASMR for both sexes, females, and males. **(D–F)** ASDR for both sexes, females, and males. Blue shading shows 5–95% quantile prediction intervals in 10% increments. Solid circles indicate observed cases. Solid lines indicate predictive means. Vertical dashed lines mark the prediction start. ASMR, age-standardized mortality rate; ASDR, age-standardized DALY rate.

The BAPC model predicted a declining trend in both ASMR and ASDR for total and ambient PM_2.5_ over the next 15 years, regardless of sex. For household PM_2.5_, the projected ASMR and ASDR over the next 15 years show an upward trend for both sexes and females, whereas ASMR in males is expected to remain stable, and ASDR is projected to decline (Figure 4; Supplementary Figures S7, S8).

## 4 Discussion

This study conducted a comprehensive, sex-specific longitudinal analysis of disease burden linked to total, ambient, and household PM_2.5_ exposure in China from 1990 to 2021. Joinpoint regression and age-period-cohort analyses showed a marked decline in total and household PM_2.5_ burdens over the past three decades. In contrast, the ambient PM_2.5_ burden in 2021, though reduced from its 2014 peak, remained well above 1990 levels. Age-specific patterns identified children and older adults as the most vulnerable groups, with household PM_2.5_ exerting the greatest impact on children and ambient PM_2.5_ on older adults. An elevated burden among younger adults (30–34 years) from ambient PM_2.5_ also merits attention. BAPC projections suggest ongoing declines in total and ambient PM_2.5_ burdens but indicate a potential rebound in household PM_2.5_ burden, particularly among females.

One of the most significant findings from this study is the divergence in disease burden trajectories across sources of PM_2.5_. In 2021, both the ASMR and ASDR for total PM_2.5_ showed a decline of over 50% compared to 2019. The net drift and AAPC results supported that this decrease was statistically significant. These findings indicate a substantial reduction in PM_2.5_ disease burden in China over the past 32 years. However, focused analysis of the disease burden and temporal trends related to ambient PM_2.5_ and household PM_2.5_ has revealed two distinct trajectories moving in opposite directions. Between 1990 and 2014, ambient PM_2.5_-attributable mortality and DALYs rose significantly, which coincided with China’s urban expansion and increased industrial output (28). For instance, during the first quarter of 2013, China experienced an exceptionally severe and prolonged haze of air pollution. Data from 74 major cities indicated that in January, 69% of the days had daily average PM_2.5_ levels surpassing the country’s air quality standard of 75 μg/m^3^, with maximum readings reaching 772 μg/m^3^ (29). This episode affected approximately 1.3 million square kilometers of land and impacted 800 million people, presenting a considerable risk to public health. In 2013, China launched the Air Pollution Prevention and Control Action Plan (APPCA), which significantly reduced ambient PM_2.5_ pollution (30, 31). The national population-weighted annual average concentration of PM_2.5_ decreased from 67.4 μg/m^3^ in 2013 to 45.5 μg/m^3^ in 2017, which equates to a 32% reduction. This reduction consequently led to a 14% reduction in premature deaths associated with long-term exposure (32). Subsequently, China implemented the “Three-Year Action Plan” in 2018, which resulted in a reduction of 0.6 teragrams in PM_2.5_ emissions by 2020, and the proportion of cities exceeding the air quality standard fell to 40.1% (33). However, in 2022, the annual average concentration of PM_2.5_ was six times higher than the 2021 World Health Organization guideline; this underscores the need for more effective strategies to improve air quality and protect public health (33).

Compared to outdoor air pollutants, indoor air pollution involves a greater number and variety of pollutants, with poor ventilation contributing to higher concentrations and more complex compositions (34). In 2017, PM_2.5_ was identified as the indoor air pollutant that contributed the most to DALYs (35). China’s rural population, the second-largest in the world, relied on biomass (mainly wood and crop residues) for about 80% of its energy consumption in 2003, and 10% of that came from coal. Although initiatives were underway to eliminate household coal usage, many cities continued to depend on coal in 2003 (36). Over the past two decades, natural gas and electric stoves have gradually replaced solid fuels for cooking (37). Heating with solid fuels has also been replaced by electric heating devices, such as heat pumps, especially in rural areas (14, 38). These changes have significantly contributed to reducing household PM_2.5_ levels. In this study, both joinpoint regression and age-period-cohort analyses consistently revealed a substantial decline in household PM_2.5_-attributable disease burden over the past three decades, underscoring the long-term benefits of China’s air pollution control policies and public health measures. However, it is important to note that this study did not include specific empirical analyses of energy consumption, fuel-switching rates, urban–rural differences, or the effectiveness of policy implementation, nor did it directly assess the relationship between these factors and the household PM_2.5_-attributable disease burden. Therefore, the observed association between the decrease in household PM_2.5_ burden and energy transition measures requires further investigation and more detailed analysis.

In this study, the age-specific disease burden attributable to ambient PM_2.5_ and household PM_2.5_ in China displayed distinct patterns across different age groups. According to age-period-cohort analysis, children under 5 years old showed markedly higher rates of mortality and DALYs from household PM_2.5_. This finding underscores the particularly high risk that household air pollution poses to young children relative to other age groups. Younger children are biologically more vulnerable due to underdeveloped lungs and immune systems. They often spend more time indoors, breathe more pollutants relative to their body mass, and have an increased risk of associated acute respiratory infections (39). In contrast, mortality and DALY rates related to ambient PM_2.5_ were relatively low among younger age groups but increased significantly in the older adults (aged 70+). This observation likely resulted from the cumulative effects of prolonged exposure to ambient air pollution.

Local drift was employed in this study as an additional measure to evaluate age-specific temporal trends in disease burden. The local drift for children under five was consistently negative and the lowest among all age groups, indicating the most rapid decline in disease burden across all PM_2.5_ sources and trends. This encouraging pattern indicates that early childhood health interventions and efforts to reduce household air pollution may have had a lasting positive effect on this vulnerable group, despite the overall increase in ambient PM_2.5_ levels during the study period (40–42). On the other end, local drift curves consistently show a peak for adults over 95 and a nearly peak value in the 30–34 age group across all PM_2.5_ sources, sexes, and indicators. Although individuals aged 30–34 are generally considered healthy with a relatively low absolute disease burden, our findings suggest that this age group may be experiencing notable relative increases in health risks. China’s rapid urbanization has led to increased motor vehicle emissions, with peak-hour traffic congestion markedly elevating ambient PM_2.5_ concentrations, thereby exacerbating associated health risks (43, 44). Moreover, occupational studies have shown that PM_2.5_ exposure in sectors such as transportation and construction contributes to increased health risks (45–47). As young adults are highly active in both work and mobility, these exposures may partly explain the rising environmental PM_2.5_ burden observed in this age group. Such shifts may be overlooked in public health planning, which tends to prioritize groups with higher absolute risk (48). However, it is important to emphasize that our results are based on age-period-cohort analyses, and further detailed research and analysis are needed to better understand the mechanisms driving these trends. The 95 + age group, representing the oldest old, is likely to have accumulated damages due to long-term PM_2.5_ exposure and age-related declines, as well as a higher burden of comorbidities (9, 49, 50). These findings emphasize the importance of recognizing the changing health risks in young adults, underscoring the need for targeted surveillance and interventions, and urging greater attention to different demographics.

In addition to age-related vulnerability, sex-based differences in PM_2.5_-related health risks have also been a subject of growing interest. In the present study, we observed that males in China consistently exhibited a higher disease burden from PM_2.5_ across all sources of exposure. Although cooking is traditionally performed by women, putting them at greater risk of exposure to household PM_2.5_, all household members are vulnerable to these adverse health effects (51). Moreover, the overall disease burden, particularly from cardiometabolic diseases, has been shown to be higher in males, which may partly explain the greater household PM_2.5_-attributable disease burden observed in males (16).

The BAPC projections from 2022 to 2036 suggest a continued decline in total and ambient PM_2.5_ burden, but a rebound in household PM_2.5_, particularly among females. This discrepancy may reflect the uneven pace of pollution control. While ambient air quality has improved through strict regulations and cleaner technologies, household pollution reduction remains inconsistent (52, 53). Significant disparities persist in residential energy consumption across rural China, where economic conditions play a crucial role in energy inequality. Low-income households continue to rely on traditional solid fuels and inefficient stoves, resulting in sustained household PM_2.5_ exposure (54, 55). Although females generally bear a lower overall PM_2.5_-attributable disease burden than males, women in low-income rural areas-who are primarily responsible for household activities-remain at elevated risk due to greater exposure to indoor pollution (56). Therefore, promoting clean energy transitions and equitable energy access is crucial to reducing household PM_2.5_ burden and related inequalities.

The study has several limitations. First, fixed PM_2.5_ monitoring in China started around 2013, and satellite data have only been available since 1998, which may lead to misclassification of early-year exposure (57). Second, GBD assessments only account for solid fuel use for cooking when estimating household air pollution; this could underestimate pollution levels in cold regions where heating also generates pollutants (58, 59). Thirdly, the wide uncertainty intervals in household PM_2.5_ estimates stem from limitations in the GBD model, including unaccounted biases and assumptions of no unmeasured confounding, particularly at high exposure levels. This underscores the need for more high-quality studies in highly polluted settings (16). Fourth, as this study relies on GBD data, it does not include mechanistic research or empirical analyses of energy use, fuel-switching rates, or policy implementation. Therefore, the observed trends are descriptive. Further research is needed to explore the underlying mechanisms.

## 5 Conclusion

In summary, despite marked improvements in air quality, PM_2.5_-related health losses in China remain unevenly distributed across populations and pollution sources. The continued dominance of ambient PM_2.5_, together with the potential resurgence of household pollution, points to the need for integrated environmental and public health strategies. Strengthening clean energy transitions, protecting vulnerable age groups, and reducing gender disparities are critical to achieving equitable and sustainable improvements in population health.

## Data Availability

The original contributions presented in the study are included in the article/Supplementary material, further inquiries can be directed to the corresponding authors.

## Supplementary material

SUPPLEMENTARY FIGURE S1 Age-stratified counts and rates of deaths and DALYs from ambient PM_2.5_ in China, 2021, by sex. **(A)** Death counts. **(B)** Death rates. **(C)** DALY counts. **(D)** DALY rates. DALY, disability-adjusted life year.

SUPPLEMENTARY FIGURE S2 Age-stratified counts and rates of deaths and DALYs from household PM_2.5_ in China, 2021, by sex. **(A)** Death counts. **(B)** Death rates. **(C)** DALY counts. **(D)** DALY rates. DALY, disability-adjusted life year.

SUPPLEMENTARY FIGURE S3 Trends in ambient PM_2.5_-attributable disease burden from 1990 to 2021. **(A–C)** ASMR for both sexes, females, and males. **(D–F)** ASDR for both sexes, females, and males. ASMR, age-standardized mortality rate; ASDR, age-standardized DALY rate; APC, annual percentage change, ^*^, *p* < 0.05.

SUPPLEMENTARY FIGURE S4 Trends in household PM_2.5_-attributable disease burden from 1990 to 2021. **(A–C)** ASMR for both sexes, females, and males. **(D–F)** ASDR for both sexes, females, and males. ASMR, age-standardized mortality rate; ASDR, age-standardized DALY rate; APC, annual percentage change, ^*^, *p* < 0.05.

SUPPLEMENTARY FIGURE S5 Age-period-cohort effects on ambient PM_2.5_-attributable disease burden. **(A)** Local drift in mortality (95% CI). The dashed horizontal lines indicate the net drift. **(B)** Mortality longitudinal age curves (95% CI). **(C)** Mortality period rate ratios vs. reference (95% CI). **(D)** Mortality cohort rate ratios vs. reference (95% CI). **(E)** Local drift in DALY (95% CI). The dashed horizontal lines indicate the net drift. **(F)** DALY longitudinal age curves (95% CI). **(G)** DALY period rate ratios vs. reference (95% CI). **(H)** DALY cohort rate ratios vs. reference (95% CI). DALY, disability-adjusted life year; CI, confidence interval.

SUPPLEMENTARY FIGURE S6 Age-period-cohort effects on household PM_2.5_-attributable disease burden. **(A)** Local drift in mortality (95% CI). The dashed horizontal lines indicate the net drift. **(B)** Mortality longitudinal age curves (95% CI). **(C)** Mortality period rate ratios vs. reference (95% CI). **(D)** Mortality cohort rate ratios vs. reference (95% CI). **(E)** Local drift in DALY (95% CI). The dashed horizontal lines indicate the net drift. **(F)** DALY longitudinal age curves (95% CI). **(G)** DALY period rate ratios vs. reference (95% CI). **(H)** DALY cohort rate ratios vs. reference (95% CI). DALY, disability-adjusted life year; CI, confidence interval.

SUPPLEMENTARY FIGURE S7 Projected ambient PM_2.5_-attributable disease burden from 1990 to 2021. **(A–C)** ASMR for both sexes, females, and males. **(D–F)** ASDR for both sexes, females, and males. Blue shading shows 5-95% quantile prediction intervals in 10% increments. Solid circles indicate observed cases. Solid lines indicate predictive means. Vertical dashed lines mark the prediction start. ASMR, age-standardized mortality rate; ASDR, age-standardized DALY rate.

SUPPLEMENTARY FIGURE S8 Projected household PM_2.5_-attributable disease burden from 1990 to 2021. **(A–C)** ASMR for both sexes, females, and males. **(D–F)** ASDR for both sexes, females, and males. Blue shading shows 5-95% quantile prediction intervals in 10% increments. Solid circles indicate observed cases. Solid lines indicate predictive means. Vertical dashed lines mark the prediction start. ASMR, age-standardized mortality rate; ASDR, age-standardized DALY rate.
