# Supplementary figures and images for: Trends and projections of PM2.5-attributable disease burden in China: a GBD 2021-based analysis
Source: Front Public Health. 2026 Jan 15;14:1684344. doi: 10.3389/fpubh.2026.1684344 (PMC12852448; doi:10.3389/fpubh.2026.1684344)

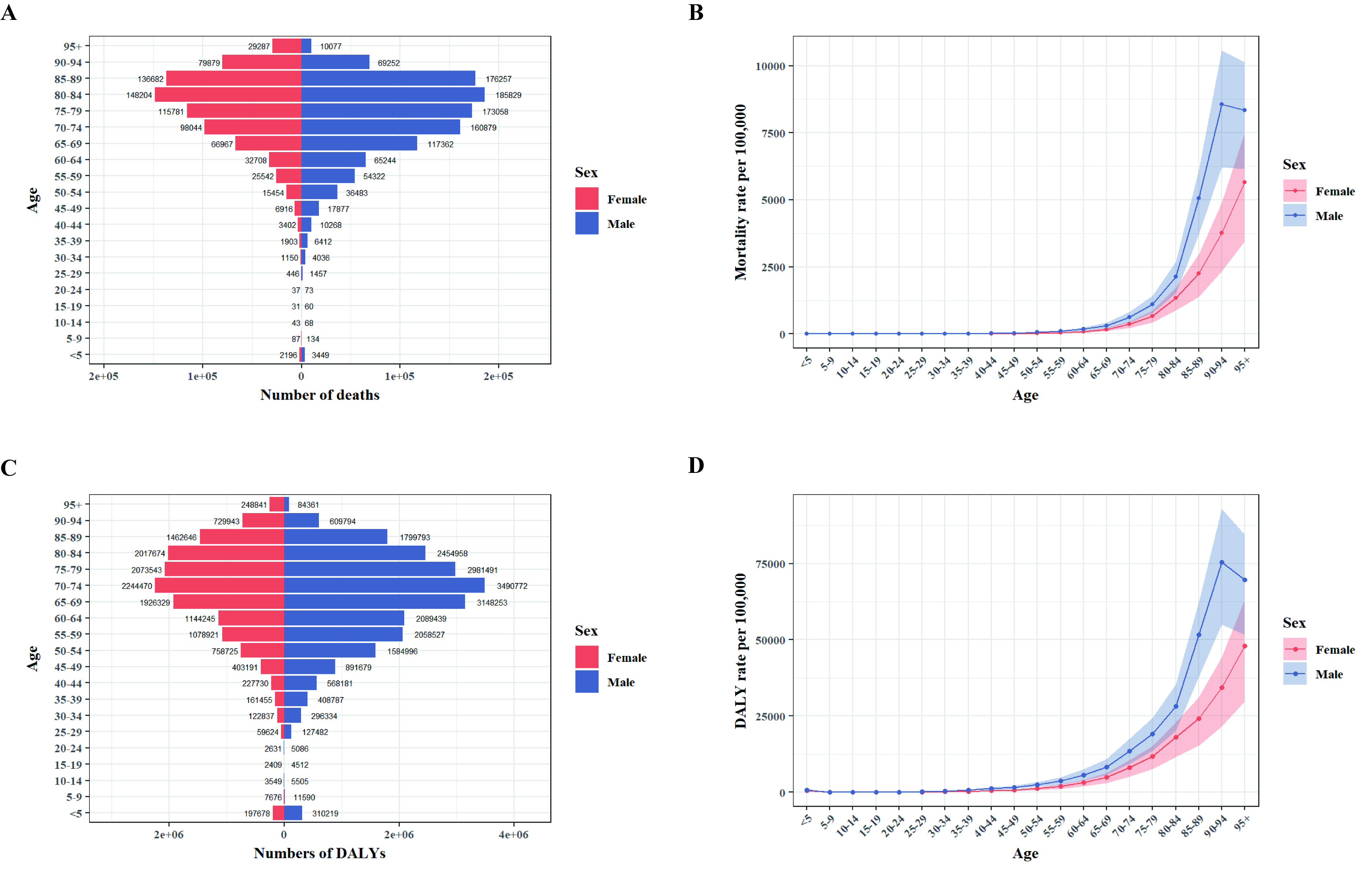

Supplement: SUPPLEMENTARY FIGURE S1 — Age-stratified counts and rates of deaths and DALYs from ambient PM2.5 in China, 2021, by sex. (A) Death counts. (B) Death rates. (C) DALY counts. (D) DALY rates. DALY, disability-adjusted life year. [file Image_1.TIF]

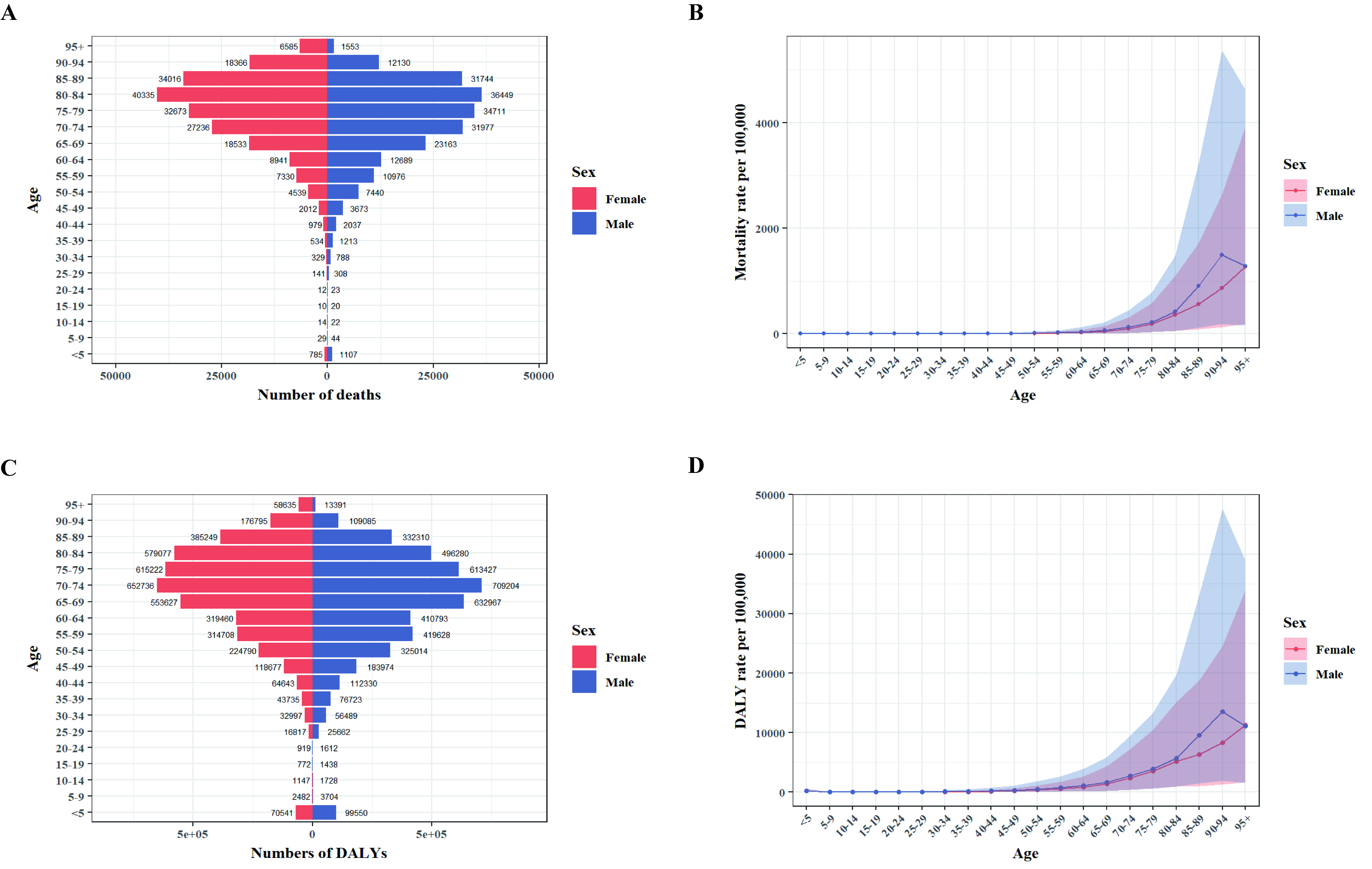

Supplement: SUPPLEMENTARY FIGURE S2 — Age-stratified counts and rates of deaths and DALYs from household PM2.5 in China, 2021, by sex. (A) Death counts. (B) Death rates. (C) DALY counts. (D) DALY rates. DALY, disability-adjusted life year. [file Image_2.TIF]

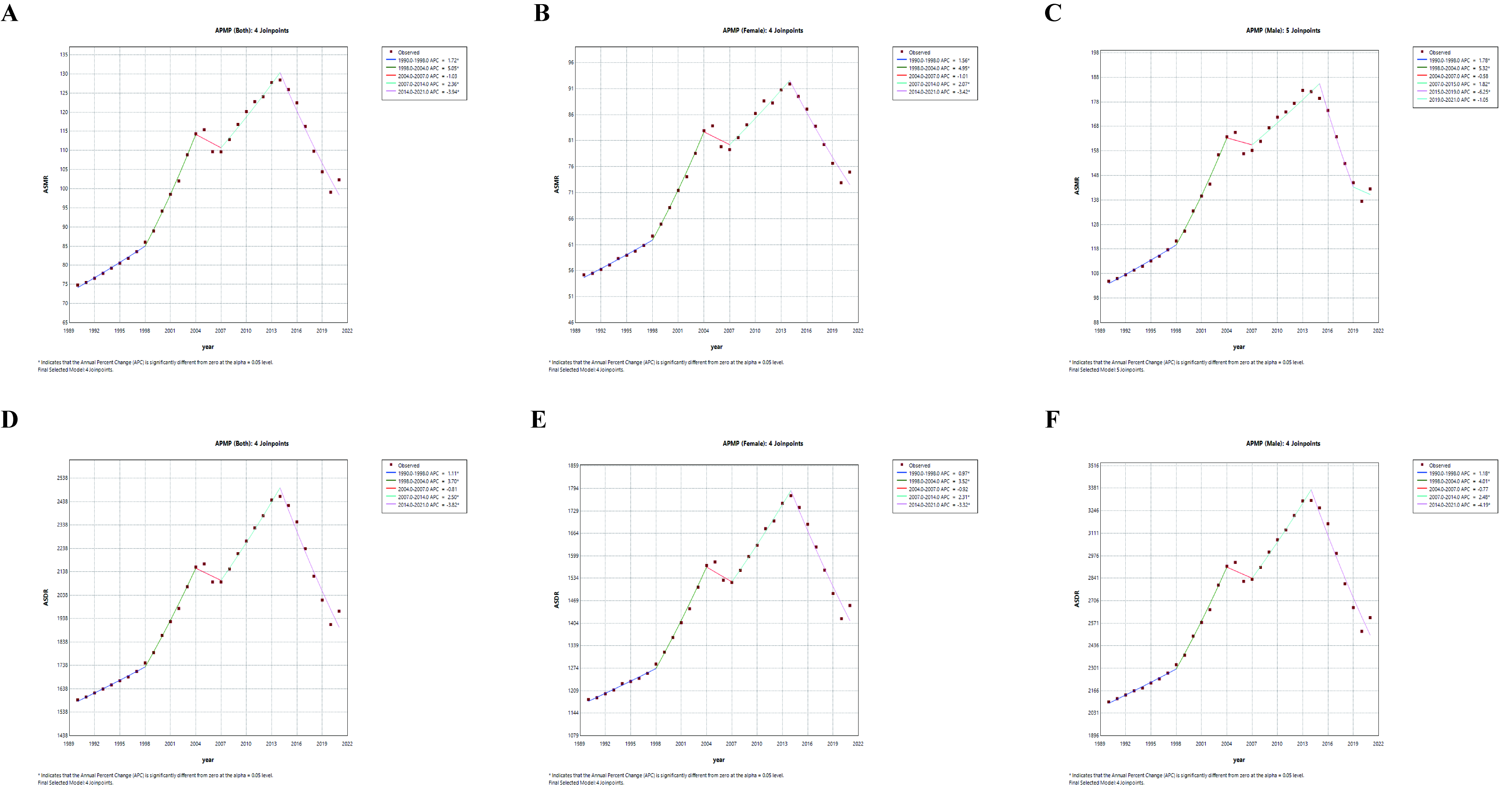

Supplement: SUPPLEMENTARY FIGURE S3 — Trends in ambient PM2.5-attributable disease burden from 1990 to 2021. (A–C) ASMR for both sexes, females, and males. (D–F) ASDR for both sexes, females, and males. ASMR, age-standardized mortality rate; ASDR, age-standardized DALY rate; APC, annual percentage change, *, p < 0.05. [file Image_3.TIF]

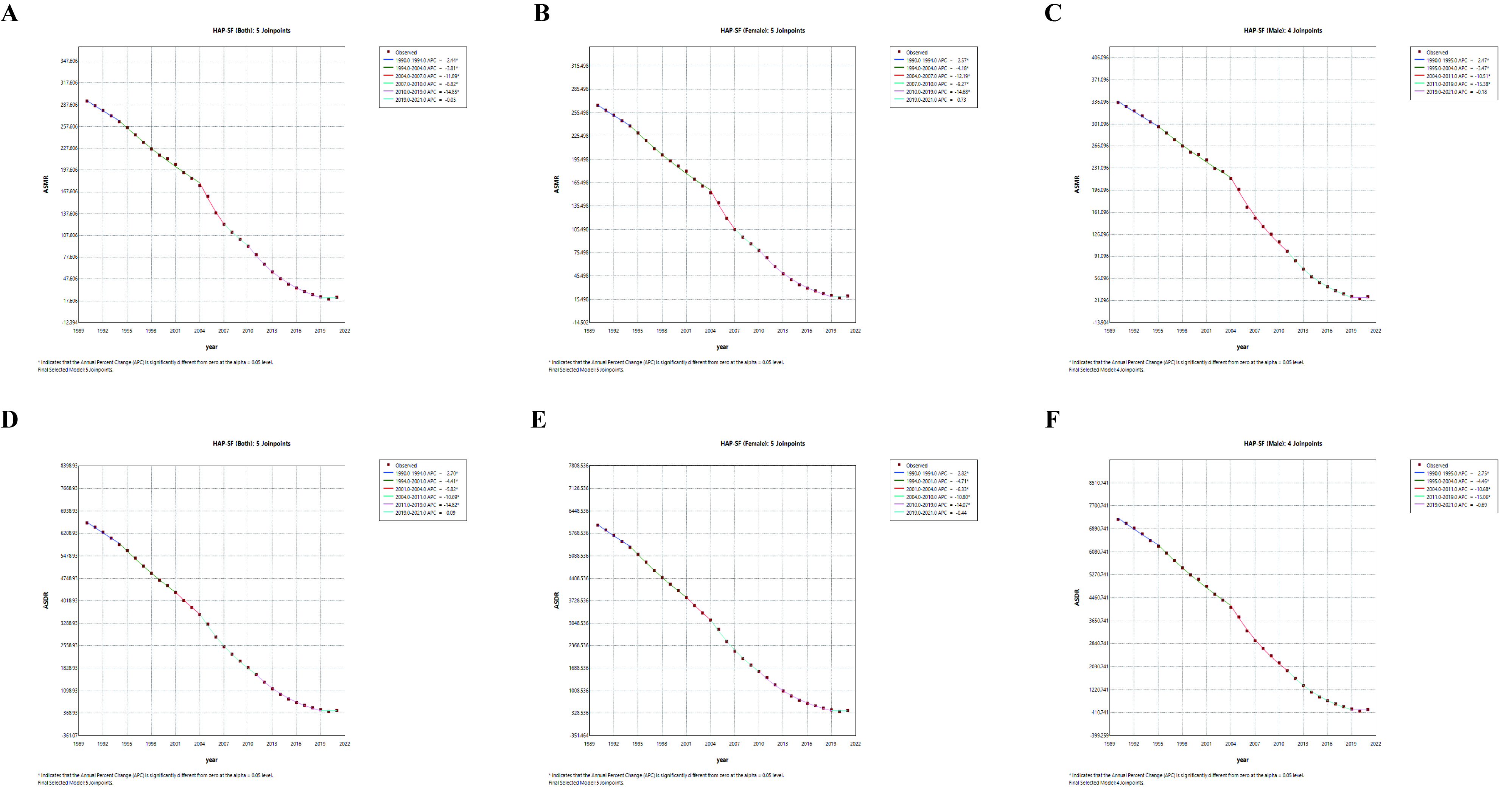

Supplement: SUPPLEMENTARY FIGURE S4 — Trends in household PM2.5-attributable disease burden from 1990 to 2021. (A–C) ASMR for both sexes, females, and males. (D–F) ASDR for both sexes, females, and males. ASMR, age-standardized mortality rate; ASDR, age-standardized DALY rate; APC, annual percentage change, *, p < 0.05. [file Image_4.TIF]

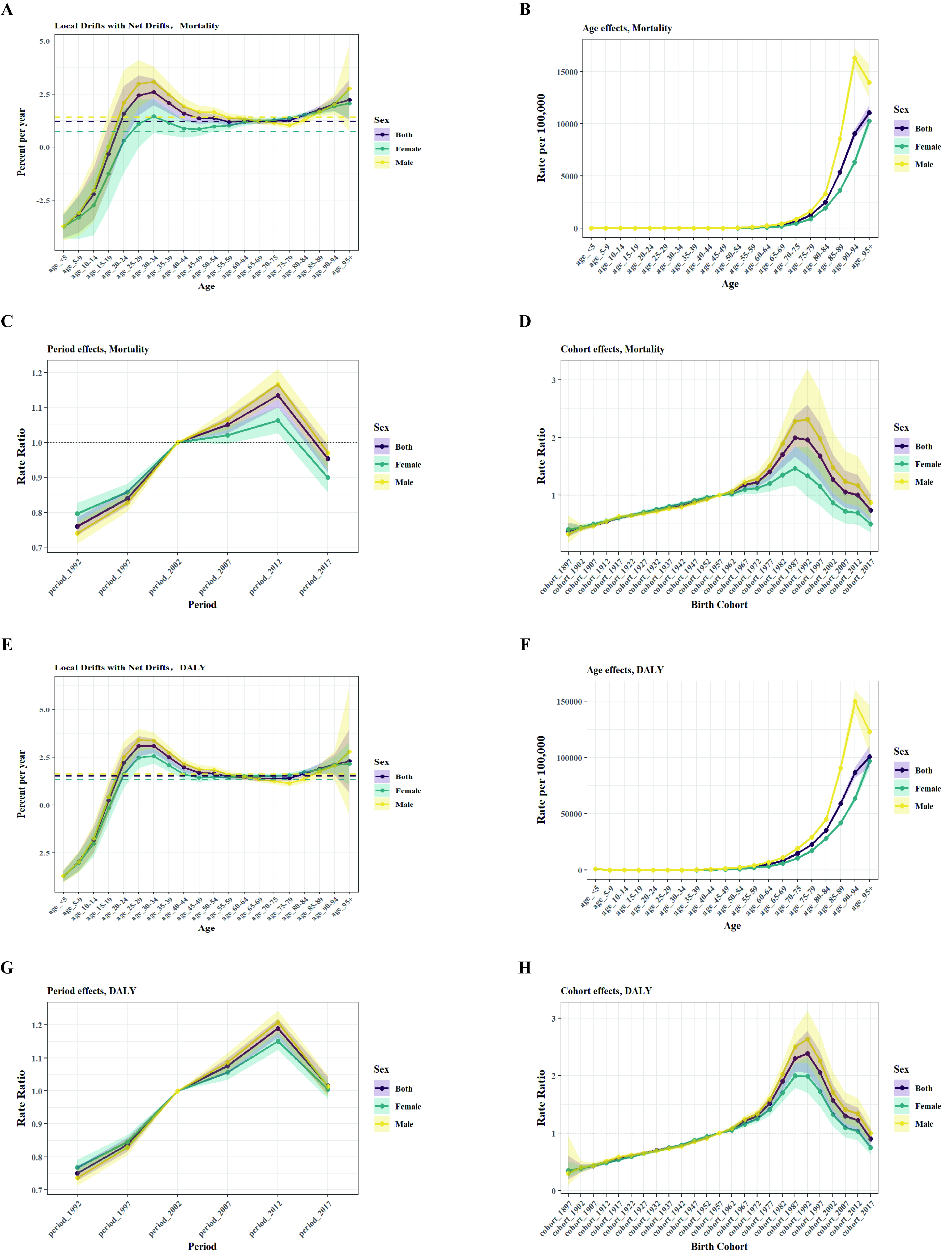

Supplement: SUPPLEMENTARY FIGURE S5 — Age-period-cohort effects on ambient PM2.5-attributable disease burden. (A) Local drift in mortality (95% CI). The dashed horizontal lines indicate the net drift. (B) Mortality longitudinal age curves (95% CI). (C) Mortality period rate ratios vs. reference (95% CI). (D) Mortality cohort rate ratios vs. reference (95% CI). (E) Local drift in DALY (95% CI). The dashed horizontal lines indicate the net drift. (F) DALY longitudinal age curves (95% CI). (G) DALY period rate ratios vs. reference (95% CI). (H) DALY cohort rate ratios vs. reference (95% CI). DALY, disability-adjusted life year; CI, confidence interval. [file Image_5.TIF]

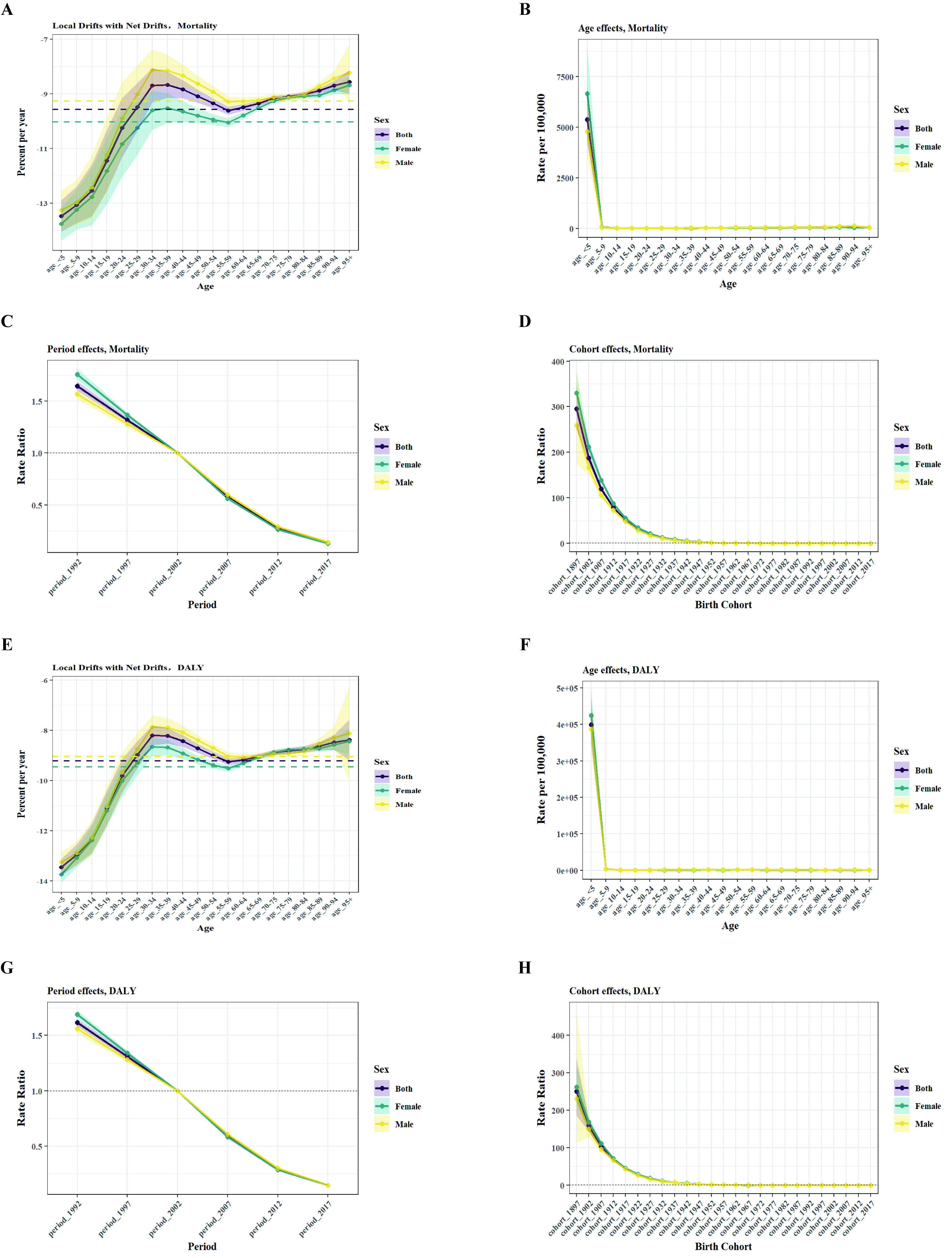

Supplement: SUPPLEMENTARY FIGURE S6 — Age-period-cohort effects on household PM2.5-attributable disease burden. (A) Local drift in mortality (95% CI). The dashed horizontal lines indicate the net drift. (B) Mortality longitudinal age curves (95% CI). (C) Mortality period rate ratios vs. reference (95% CI). (D) Mortality cohort rate ratios vs. reference (95% CI). (E) Local drift in DALY (95% CI). The dashed horizontal lines indicate the net drift. (F) DALY longitudinal age curves (95% CI). (G) DALY period rate ratios vs. reference (95% CI). (H) DALY cohort rate ratios vs. reference (95% CI). DALY, disability-adjusted life year; CI, confidence interval. [file Image_6.TIF]

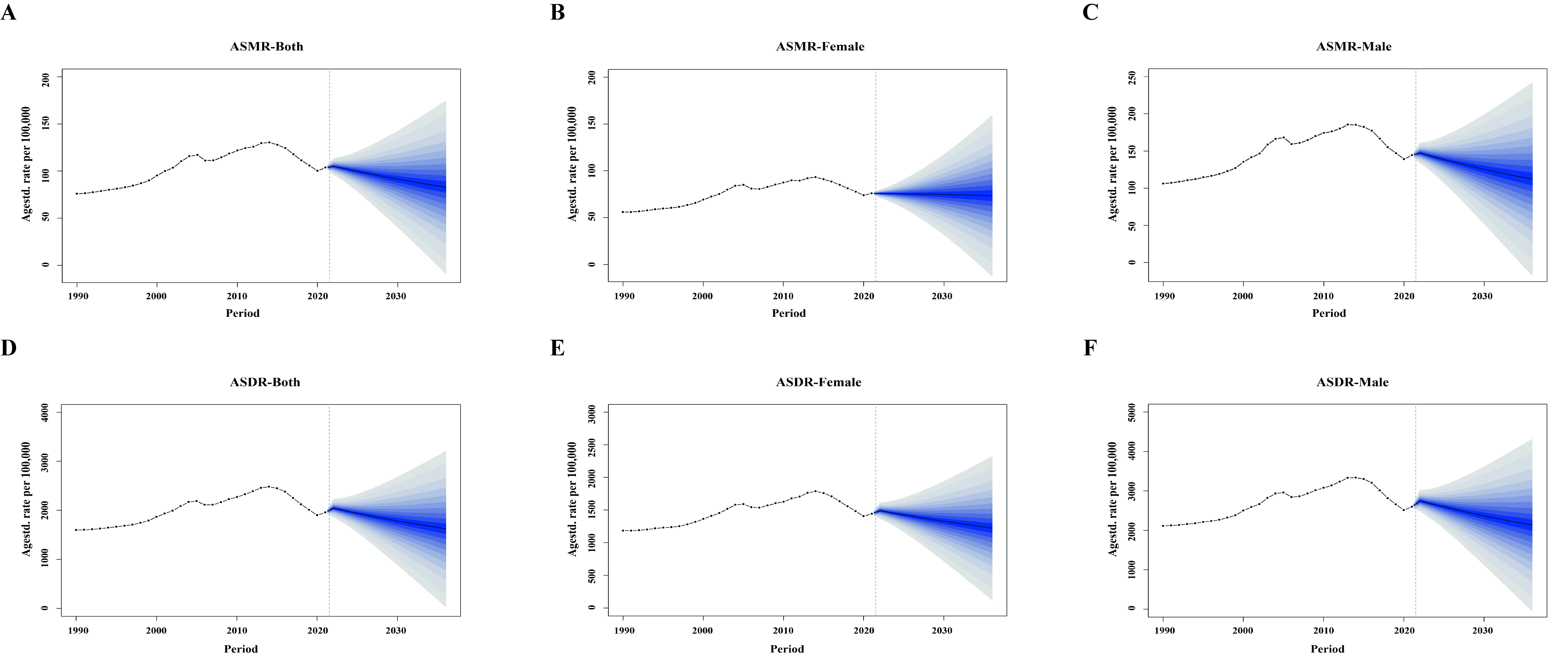

Supplement: SUPPLEMENTARY FIGURE S7 — Projected ambient PM2.5-attributable disease burden from 1990 to 2021. (A–C) ASMR for both sexes, females, and males. (D–F) ASDR for both sexes, females, and males. Blue shading shows 5-95% quantile prediction intervals in 10% increments. Solid circles indicate observed cases. Solid lines indicate predictive means. Vertical dashed lines mark the prediction start. ASMR, age-standardized mortality rate; ASDR, age-standardized DALY rate. [file Image_7.TIF]

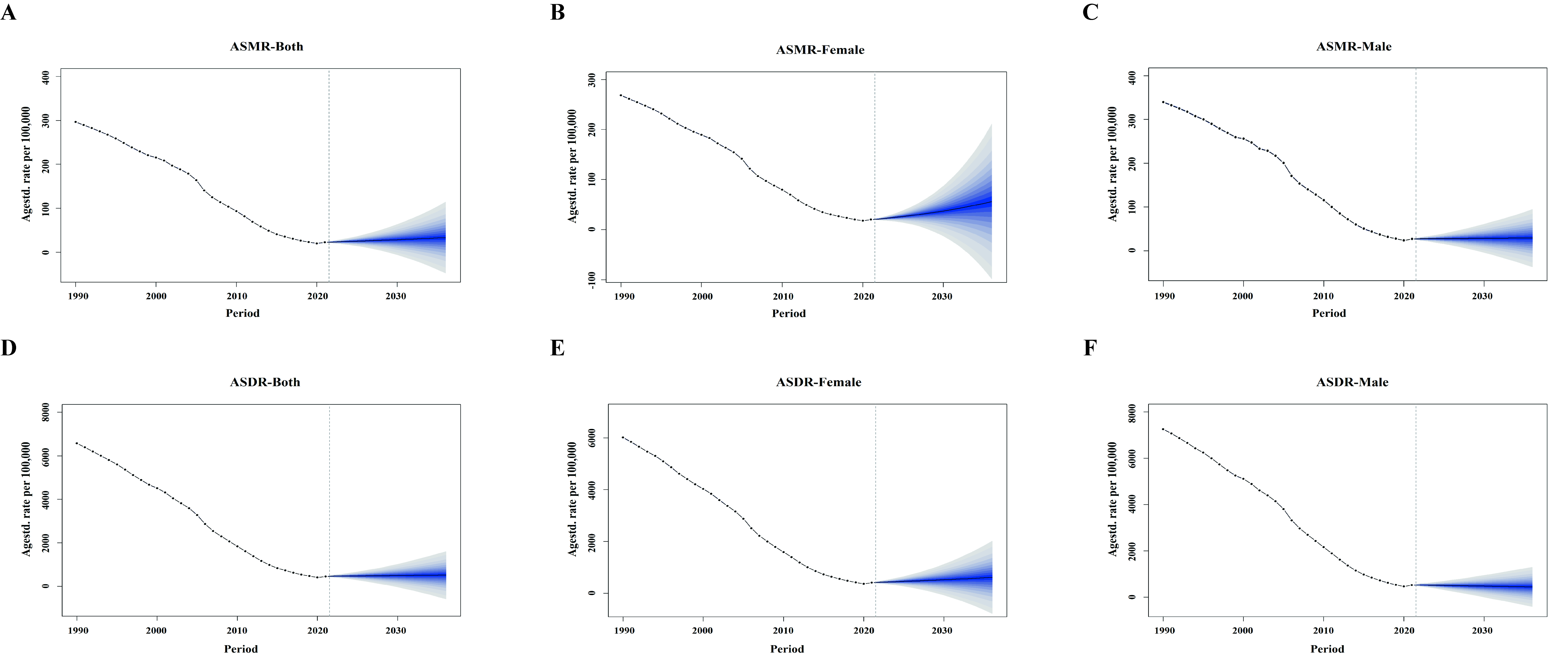

Supplement: SUPPLEMENTARY FIGURE S8 — Projected household PM2.5-attributable disease burden from 1990 to 2021. (A–C) ASMR for both sexes, females, and males. (D–F) ASDR for both sexes, females, and males. Blue shading shows 5-95% quantile prediction intervals in 10% increments. Solid circles indicate observed cases. Solid lines indicate predictive means. Vertical dashed lines mark the prediction start. ASMR, age-standardized mortality rate; ASDR, age-standardized DALY rate. [file Image_8.TIF]
