# Supplementary material for: Trends and projections of PM2.5-attributable disease burden in China: a GBD 2021-based analysis
Source: Front Public Health. 2026 Jan 15;14:1684344. doi: 10.3389/fpubh.2026.1684344 (PMC12852448; doi:10.3389/fpubh.2026.1684344)
Supplement: Supplementary file 9 [file Table_1.DOCX]

| **Table S1. Time trends of PM_2.5_-attributable DALYs in China, 1990–2021, stratified by sex** | | | | | | |
| --- | --- | --- | --- | --- | --- | --- |
| **Characteristics** | **1990** | | **2021** | | **1990-2021** | **Net drift^†^** |
|  | **All-ages cases(million)**  **n(95%UI)** | **ASMR per 100,000**  **n(95%UI)** | **All-ages cases(million)**  **n(95%UI)** | **ASMR per 100,000**  **n(95%UI)** | **AAPC in ASMR**  **(95%CI)** | **% per year** |
| **PMP** |  |  |  |  |  |  |
| Both | 66.32  (53.46-77.46) | 8,142.70  (6,796.79-9,441.41) | 46.68  (36.58-59.74) | 2,436.88  (1,919.69-3,069.45) | -3.93^*^  (-4.22 - -3.65) | -3.87^*^  (-4.01 - -3.72) |
| Female | 29.80  (23.54-35.38) | 7,199.51  (5,780.16-8,473.09) | 19.11  (14.68-23.92) | 1,872.04  (1,458.83-2,327.44) | -4.31^*^  (-4.58 - -4.03) | -4.60^*^  (-4.73 - -4.47) |
| Male | 36.52  (29.45-43.89) | 9,320.54  (7,739.13-11,087.93) | 27.56  (20.90-36.35) | 3,132.22  (2,419.03-4,065.36) | -3.54^*^  (-3.86 - -3.22) | -3.39^*^  (-3.56 - -3.21) |
| **APMP** |  |  |  |  |  |  |
| Both | 12.73  (6.16-22.70) | 1,590.95  (768.20-2,818.49) | 37.81  (26.28-46.52) | 1,970.10  (1,373.00-2,423.21) | 0.59  (-0.06 - 1.24) | 1.52^*^  (1.37 - 1.67) |
| Female | 4.82  (2.20-8.56) | 1,183.48  (542.39-2,104.27) | 14.87  (9.41-19.25) | 1,455.28  (915.76-1,878.68) | 0.58^*^  (0.08 - 1.09) | 1.33^*^  (1.18 - 1.49) |
| Male | 7.91  (3.78-14.35) | 2,098.85  (1,011.87-3,769.54) | 22.93  (16.13-29.42) | 2,604.27  (1,840.54-3,286.06) | 0.58  (-0.13 - 1.29) | 1.62^*^  (1.43 - 1.80) |
| **HAP-SF** |  |  |  |  |  |  |
| Both | 53.59  (40.75-64.92) | 6,551.36  (5,073.76-7,895.68) | 8.86  (1.49-27.82) | 466.10  (81.56-1,455.82) | -8.28^*^  (-8.93 - -7.62) | -9.22^*^  (-9.37 - -9.08) |
| Female | 24.98  (18.76-30.41) | 6,015.62  (4,596.91-7,287.44) | 4.23  (0.741-12.86) | 416.22  (75.35-1,246.96) | -8.37^*^  (-8.96 - -7.77) | -9.45^*^  (-9.59 - -9.31) |
| Male | 28.61  (21.15-35.85) | 7,221.32  (5,379.86-9,016.62) | 4.63  (0.72-15.77) | 527.08  (84.98-1,807.19) | -8.23^*^  (-9.01 - -7.43) | -9.05^*^  (-9.23 - -8.88) |
| ASDR, age-standardized DALY rate; UI, uncertainty interval; AAPC, average annual percentage change; CI, confidential interval; *, *p* < 0.05.  † Net drifts are estimates derived from the age-period-cohort model and denotes overall annual percent change in DALYs, with values expressed as percent change per year (% per year). | | | | | | |
