# Supplementary material for: Trends and projections of PM2.5-attributable disease burden in China: a GBD 2021-based analysis
Source: Front Public Health. 2026 Jan 15;14:1684344. doi: 10.3389/fpubh.2026.1684344 (PMC12852448; doi:10.3389/fpubh.2026.1684344)
Supplement: Supplementary file 10 [file Table_2.DOCX]

| **Table S2. Annual percentage change in PMP-attributable mortality and DALYs by age group and sex, 1990-2021 (local drift)** | | | | | |  |
| --- | --- | --- | --- | --- | --- | --- |
| **Measure** | **Age** | **Sex** | **Percent per Year** | **95%CI_Low** | **95%CI_High** | |
| Mortality | age_<5 | Both | -9.3802 | -9.8701 | -8.8876 | |
| Mortality | age_5-9 | Both | -8.7268 | -9.4488 | -7.9991 | |
| Mortality | age_10-14 | Both | -7.7037 | -8.714 | -6.6822 | |
| Mortality | age_15-19 | Both | -6.0114 | -7.1541 | -4.8547 | |
| Mortality | age_20-24 | Both | -4.393 | -5.4688 | -3.3049 | |
| Mortality | age_25-29 | Both | -3.5241 | -4.3353 | -2.7061 | |
| Mortality | age_30-34 | Both | -2.9358 | -3.4967 | -2.3718 | |
| Mortality | age_35-39 | Both | -3.2221 | -3.6508 | -2.7915 | |
| Mortality | age_40-44 | Both | -3.5639 | -3.8747 | -3.252 | |
| Mortality | age_45-49 | Both | -3.8071 | -4.0334 | -3.5803 | |
| Mortality | age_50-54 | Both | -3.9533 | -4.1271 | -3.7792 | |
| Mortality | age_55-59 | Both | -4.2017 | -4.3465 | -4.0567 | |
| Mortality | age_60-64 | Both | -4.1403 | -4.258 | -4.0225 | |
| Mortality | age_65-69 | Both | -4.0752 | -4.1722 | -3.978 | |
| Mortality | age_70-75 | Both | -3.9359 | -4.0219 | -3.8499 | |
| Mortality | age_75-79 | Both | -3.8328 | -3.9139 | -3.7515 | |
| Mortality | age_80-84 | Both | -3.6169 | -3.7034 | -3.5303 | |
| Mortality | age_85-89 | Both | -3.373 | -3.4873 | -3.2585 | |
| Mortality | age_90-94 | Both | -3.1448 | -3.35 | -2.9391 | |
| Mortality | age_95+ | Both | -3.029 | -3.598 | -2.4566 | |
| Mortality | age_<5 | Female | -9.744 | -10.2366 | -9.2487 | |
| Mortality | age_5-9 | Female | -9.0797 | -9.8385 | -8.3146 | |
| Mortality | age_10-14 | Female | -8.3335 | -9.4142 | -7.24 | |
| Mortality | age_15-19 | Female | -7.0385 | -8.2726 | -5.7878 | |
| Mortality | age_20-24 | Female | -5.844 | -7.0187 | -4.6544 | |
| Mortality | age_25-29 | Female | -5.2205 | -6.1292 | -4.3029 | |
| Mortality | age_30-34 | Female | -4.696 | -5.3421 | -4.0455 | |
| Mortality | age_35-39 | Female | -4.8043 | -5.2986 | -4.3075 | |
| Mortality | age_40-44 | Female | -4.9816 | -5.3342 | -4.6277 | |
| Mortality | age_45-49 | Female | -5.0658 | -5.3162 | -4.8148 | |
| Mortality | age_50-54 | Female | -5.0952 | -5.283 | -4.907 | |
| Mortality | age_55-59 | Female | -5.1398 | -5.2936 | -4.9858 | |
| Mortality | age_60-64 | Female | -4.9073 | -5.03 | -4.7844 | |
| Mortality | age_65-69 | Female | -4.6959 | -4.7944 | -4.5974 | |
| Mortality | age_70-75 | Female | -4.4689 | -4.5537 | -4.384 | |
| Mortality | age_75-79 | Female | -4.2779 | -4.3548 | -4.2009 | |
| Mortality | age_80-84 | Female | -4.0679 | -4.1455 | -3.9902 | |
| Mortality | age_85-89 | Female | -3.902 | -4.0003 | -3.8035 | |
| Mortality | age_90-94 | Female | -3.6173 | -3.7841 | -3.4502 | |
| Mortality | age_95+ | Female | -3.4265 | -3.8252 | -3.0261 | |
| Mortality | age_<5 | Male | -9.1261 | -9.7787 | -8.4688 | |
| Mortality | age_5-9 | Male | -8.4999 | -9.4377 | -7.5524 | |
| Mortality | age_10-14 | Male | -7.3441 | -8.6443 | -6.0254 | |
| Mortality | age_15-19 | Male | -5.4675 | -6.9314 | -3.9806 | |
| Mortality | age_20-24 | Male | -3.6502 | -5.0223 | -2.2583 | |
| Mortality | age_25-29 | Male | -2.6877 | -3.7107 | -1.6538 | |
| Mortality | age_30-34 | Male | -2.0784 | -2.7766 | -1.3751 | |
| Mortality | age_35-39 | Male | -2.4382 | -2.9712 | -1.9022 | |
| Mortality | age_40-44 | Male | -2.8316 | -3.2209 | -2.4408 | |
| Mortality | age_45-49 | Male | -3.1113 | -3.3981 | -2.8238 | |
| Mortality | age_50-54 | Male | -3.2681 | -3.491 | -3.0447 | |
| Mortality | age_55-59 | Male | -3.6005 | -3.7878 | -3.4129 | |
| Mortality | age_60-64 | Male | -3.6292 | -3.783 | -3.4751 | |
| Mortality | age_65-69 | Male | -3.6492 | -3.7782 | -3.5199 | |
| Mortality | age_70-75 | Male | -3.595 | -3.7117 | -3.4782 | |
| Mortality | age_75-79 | Male | -3.5878 | -3.7017 | -3.4737 | |
| Mortality | age_80-84 | Male | -3.3378 | -3.4654 | -3.21 | |
| Mortality | age_85-89 | Male | -2.9363 | -3.1128 | -2.7594 | |
| Mortality | age_90-94 | Male | -2.5929 | -2.9351 | -2.2495 | |
| Mortality | age_95+ | Male | -1.9043 | -3.3504 | -0.4365 | |
| DALYs | age_<5 | Both | -9.3623 | -9.6597 | -9.0639 | |
| DALYs | age_5-9 | Both | -8.5856 | -9.0361 | -8.1328 | |
| DALYs | age_10-14 | Both | -7.3808 | -8.017 | -6.7403 | |
| DALYs | age_15-19 | Both | -5.5247 | -6.2457 | -4.7981 | |
| DALYs | age_20-24 | Both | -3.8185 | -4.4979 | -3.1343 | |
| DALYs | age_25-29 | Both | -2.9108 | -3.4274 | -2.3915 | |
| DALYs | age_30-34 | Both | -2.439 | -2.8113 | -2.0652 | |
| DALYs | age_35-39 | Both | -2.7907 | -3.0924 | -2.488 | |
| DALYs | age_40-44 | Both | -3.1879 | -3.4217 | -2.9536 | |
| DALYs | age_45-49 | Both | -3.4744 | -3.657 | -3.2915 | |
| DALYs | age_50-54 | Both | -3.6551 | -3.8065 | -3.5035 | |
| DALYs | age_55-59 | Both | -3.8959 | -4.0305 | -3.7612 | |
| DALYs | age_60-64 | Both | -3.8925 | -4.0111 | -3.7737 | |
| DALYs | age_65-69 | Both | -3.8538 | -3.9615 | -3.746 | |
| DALYs | age_70-75 | Both | -3.7521 | -3.858 | -3.6461 | |
| DALYs | age_75-79 | Both | -3.6708 | -3.784 | -3.5575 | |
| DALYs | age_80-84 | Both | -3.4816 | -3.6193 | -3.3437 | |
| DALYs | age_85-89 | Both | -3.247 | -3.4489 | -3.0446 | |
| DALYs | age_90-94 | Both | -3.0469 | -3.4368 | -2.6554 | |
| DALYs | age_95+ | Both | -2.9674 | -4.0853 | -1.8366 | |
| DALYs | age_<5 | Female | -9.7223 | -9.9875 | -9.4563 | |
| DALYs | age_5-9 | Female | -8.8811 | -9.289 | -8.4715 | |
| DALYs | age_10-14 | Female | -7.8046 | -8.3844 | -7.2212 | |
| DALYs | age_15-19 | Female | -6.1589 | -6.8182 | -5.4949 | |
| DALYs | age_20-24 | Female | -4.6974 | -5.3206 | -4.0701 | |
| DALYs | age_25-29 | Female | -3.9459 | -4.4248 | -3.4646 | |
| DALYs | age_30-34 | Female | -3.5766 | -3.9272 | -3.2248 | |
| DALYs | age_35-39 | Female | -3.8796 | -4.1666 | -3.5917 | |
| DALYs | age_40-44 | Female | -4.2323 | -4.4543 | -4.0099 | |
| DALYs | age_45-49 | Female | -4.4468 | -4.6182 | -4.2751 | |
| DALYs | age_50-54 | Female | -4.5669 | -4.7071 | -4.4265 | |
| DALYs | age_55-59 | Female | -4.6512 | -4.7743 | -4.528 | |
| DALYs | age_60-64 | Female | -4.5096 | -4.6167 | -4.4024 | |
| DALYs | age_65-69 | Female | -4.3472 | -4.4421 | -4.2522 | |
| DALYs | age_70-75 | Female | -4.1817 | -4.2725 | -4.0908 | |
| DALYs | age_75-79 | Female | -4.0397 | -4.1331 | -3.9462 | |
| DALYs | age_80-84 | Female | -3.8627 | -3.971 | -3.7542 | |
| DALYs | age_85-89 | Female | -3.7175 | -3.8703 | -3.5644 | |
| DALYs | age_90-94 | Female | -3.4698 | -3.7495 | -3.1893 | |
| DALYs | age_95+ | Female | -3.3311 | -4.0247 | -2.6325 | |
| DALYs | age_<5 | Male | -9.1111 | -9.4692 | -8.7515 | |
| DALYs | age_5-9 | Male | -8.3964 | -8.9338 | -7.8558 | |
| DALYs | age_10-14 | Male | -7.1194 | -7.8753 | -6.3572 | |
| DALYs | age_15-19 | Male | -5.1348 | -5.9902 | -4.2715 | |
| DALYs | age_20-24 | Male | -3.2781 | -4.083 | -2.4664 | |
| DALYs | age_25-29 | Male | -2.2854 | -2.8941 | -1.673 | |
| DALYs | age_30-34 | Male | -1.76 | -2.195 | -1.323 | |
| DALYs | age_35-39 | Male | -2.1514 | -2.5018 | -1.7997 | |
| DALYs | age_40-44 | Male | -2.5755 | -2.8473 | -2.303 | |
| DALYs | age_45-49 | Male | -2.8819 | -3.0956 | -2.6678 | |
| DALYs | age_50-54 | Male | -3.063 | -3.2416 | -2.8841 | |
| DALYs | age_55-59 | Male | -3.3769 | -3.5367 | -3.2168 | |
| DALYs | age_60-64 | Male | -3.4569 | -3.599 | -3.3145 | |
| DALYs | age_65-69 | Male | -3.5023 | -3.6333 | -3.3711 | |
| DALYs | age_70-75 | Male | -3.474 | -3.6056 | -3.3422 | |
| DALYs | age_75-79 | Male | -3.4656 | -3.611 | -3.3199 | |
| DALYs | age_80-84 | Male | -3.2415 | -3.427 | -3.0557 | |
| DALYs | age_85-89 | Male | -2.8459 | -3.1297 | -2.5613 | |
| DALYs | age_90-94 | Male | -2.5379 | -3.1285 | -1.9437 | |
| DALYs | age_95+ | Male | -1.8869 | -4.4608 | 0.7564 | |
