# Supplementary material for: Trends and projections of PM2.5-attributable disease burden in China: a GBD 2021-based analysis
Source: Front Public Health. 2026 Jan 15;14:1684344. doi: 10.3389/fpubh.2026.1684344 (PMC12852448; doi:10.3389/fpubh.2026.1684344)
Supplement: Supplementary file 11 [file Table_3.DOCX]

| **Table S3. Annual percentage change in APMP-attributable mortality and DALYs by age group and sex, 1990-2021 (local drift)** | | | | | |
| --- | --- | --- | --- | --- | --- |
| **Measure** | **Age** | **Sex** | **Percent per Year** | **95%CI_Low** | **95%CI_High** |
| Mortality | age_<5 | Both | -3.729 | -4.27 | -3.1849 |
| Mortality | age_5-9 | Both | -3.1516 | -4.0382 | -2.2569 |
| Mortality | age_10-14 | Both | -2.2148 | -3.4509 | -0.9628 |
| Mortality | age_15-19 | Both | -0.3204 | -1.703 | 1.0817 |
| Mortality | age_20-24 | Both | 1.5838 | 0.3015 | 2.8825 |
| Mortality | age_25-29 | Both | 2.4417 | 1.5059 | 3.3862 |
| Mortality | age_30-34 | Both | 2.593 | 1.9658 | 3.2241 |
| Mortality | age_35-39 | Both | 2.0657 | 1.5932 | 2.5404 |
| Mortality | age_40-44 | Both | 1.5786 | 1.2301 | 1.9282 |
| Mortality | age_45-49 | Both | 1.352 | 1.0907 | 1.614 |
| Mortality | age_50-54 | Both | 1.3635 | 1.158 | 1.5695 |
| Mortality | age_55-59 | Both | 1.1912 | 1.0187 | 1.3639 |
| Mortality | age_60-64 | Both | 1.2268 | 1.0839 | 1.3699 |
| Mortality | age_65-69 | Both | 1.2071 | 1.0864 | 1.3279 |
| Mortality | age_70-75 | Both | 1.2314 | 1.1222 | 1.3407 |
| Mortality | age_75-79 | Both | 1.2626 | 1.1562 | 1.3691 |
| Mortality | age_80-84 | Both | 1.5041 | 1.3863 | 1.622 |
| Mortality | age_85-89 | Both | 1.7756 | 1.6104 | 1.9412 |
| Mortality | age_90-94 | Both | 2.024 | 1.7042 | 2.3449 |
| Mortality | age_95+ | Both | 2.2298 | 1.2788 | 3.1898 |
| Mortality | age_<5 | Female | -3.7379 | -4.3017 | -3.1708 |
| Mortality | age_5-9 | Female | -3.3249 | -4.3232 | -2.3162 |
| Mortality | age_10-14 | Female | -2.7405 | -4.1529 | -1.3073 |
| Mortality | age_15-19 | Female | -1.2489 | -2.842 | 0.3704 |
| Mortality | age_20-24 | Female | 0.3052 | -1.1892 | 1.8222 |
| Mortality | age_25-29 | Female | 1.1037 | -0.0196 | 2.2395 |
| Mortality | age_30-34 | Female | 1.4481 | 0.6676 | 2.2346 |
| Mortality | age_35-39 | Female | 1.1475 | 0.5575 | 1.741 |
| Mortality | age_40-44 | Female | 0.8757 | 0.4488 | 1.3044 |
| Mortality | age_45-49 | Female | 0.8412 | 0.5295 | 1.154 |
| Mortality | age_50-54 | Female | 0.969 | 0.7297 | 1.2088 |
| Mortality | age_55-59 | Female | 1.0114 | 0.8143 | 1.2089 |
| Mortality | age_60-64 | Female | 1.1755 | 1.0151 | 1.3361 |
| Mortality | age_65-69 | Female | 1.2347 | 1.103 | 1.3665 |
| Mortality | age_70-75 | Female | 1.2852 | 1.1696 | 1.4009 |
| Mortality | age_75-79 | Female | 1.3615 | 1.2533 | 1.4698 |
| Mortality | age_80-84 | Female | 1.5192 | 1.4064 | 1.6321 |
| Mortality | age_85-89 | Female | 1.6772 | 1.5265 | 1.8281 |
| Mortality | age_90-94 | Female | 1.9329 | 1.657 | 2.2096 |
| Mortality | age_95+ | Female | 2.0502 | 1.3522 | 2.7531 |
| Mortality | age_<5 | Male | -3.7204 | -4.4007 | -3.0352 |
| Mortality | age_5-9 | Male | -3.1102 | -4.1828 | -2.0256 |
| Mortality | age_10-14 | Male | -2.0324 | -3.515 | -0.5271 |
| Mortality | age_15-19 | Male | 0.0337 | -1.6174 | 1.7124 |
| Mortality | age_20-24 | Male | 2.1055 | 0.5815 | 3.6525 |
| Mortality | age_25-29 | Male | 2.9921 | 1.8937 | 4.1023 |
| Mortality | age_30-34 | Male | 3.0705 | 2.345 | 3.8012 |
| Mortality | age_35-39 | Male | 2.4736 | 1.9276 | 3.0225 |
| Mortality | age_40-44 | Male | 1.9231 | 1.5175 | 2.3303 |
| Mortality | age_45-49 | Male | 1.6486 | 1.3411 | 1.9571 |
| Mortality | age_50-54 | Male | 1.6458 | 1.4013 | 1.891 |
| Mortality | age_55-59 | Male | 1.3827 | 1.1758 | 1.5901 |
| Mortality | age_60-64 | Male | 1.3323 | 1.1592 | 1.5056 |
| Mortality | age_65-69 | Male | 1.23 | 1.0819 | 1.3784 |
| Mortality | age_70-75 | Male | 1.1482 | 1.0116 | 1.2849 |
| Mortality | age_75-79 | Male | 1.0298 | 0.8931 | 1.1667 |
| Mortality | age_80-84 | Male | 1.2749 | 1.1165 | 1.4336 |
| Mortality | age_85-89 | Male | 1.6679 | 1.436 | 1.9003 |
| Mortality | age_90-94 | Male | 2.0091 | 1.531 | 2.4895 |
| Mortality | age_95+ | Male | 2.7664 | 0.7248 | 4.8494 |
| DALYs | age_<5 | Both | -3.7098 | -3.9995 | -3.4192 |
| DALYs | age_5-9 | Both | -2.9576 | -3.4418 | -2.4711 |
| DALYs | age_10-14 | Both | -1.8101 | -2.4897 | -1.1258 |
| DALYs | age_15-19 | Both | 0.2525 | -0.5103 | 1.0211 |
| DALYs | age_20-24 | Both | 2.2179 | 1.5079 | 2.9329 |
| DALYs | age_25-29 | Both | 3.0864 | 2.5595 | 3.616 |
| DALYs | age_30-34 | Both | 3.0879 | 2.7136 | 3.4635 |
| DALYs | age_35-39 | Both | 2.5058 | 2.2047 | 2.8077 |
| DALYs | age_40-44 | Both | 1.9614 | 1.7229 | 2.2005 |
| DALYs | age_45-49 | Both | 1.6857 | 1.4933 | 1.8785 |
| DALYs | age_50-54 | Both | 1.6593 | 1.4958 | 1.8232 |
| DALYs | age_55-59 | Both | 1.4881 | 1.3414 | 1.6351 |
| DALYs | age_60-64 | Both | 1.4504 | 1.3189 | 1.5821 |
| DALYs | age_65-69 | Both | 1.3948 | 1.2735 | 1.5163 |
| DALYs | age_70-75 | Both | 1.386 | 1.2651 | 1.507 |
| DALYs | age_75-79 | Both | 1.4035 | 1.2706 | 1.5366 |
| DALYs | age_80-84 | Both | 1.6357 | 1.4662 | 1.8055 |
| DALYs | age_85-89 | Both | 1.9101 | 1.6457 | 2.1753 |
| DALYs | age_90-94 | Both | 2.1285 | 1.5857 | 2.6741 |
| DALYs | age_95+ | Both | 2.2937 | 0.6397 | 3.9748 |
| DALYs | age_<5 | Female | -3.718 | -3.9989 | -3.4362 |
| DALYs | age_5-9 | Female | -3.0062 | -3.4906 | -2.5194 |
| DALYs | age_10-14 | Female | -2.0176 | -2.7018 | -1.3286 |
| DALYs | age_15-19 | Female | -0.157 | -0.9279 | 0.6198 |
| DALYs | age_20-24 | Female | 1.6225 | 0.9007 | 2.3494 |
| DALYs | age_25-29 | Female | 2.4801 | 1.9345 | 3.0286 |
| DALYs | age_30-34 | Female | 2.566 | 2.1672 | 2.9663 |
| DALYs | age_35-39 | Female | 2.0705 | 1.745 | 2.397 |
| DALYs | age_40-44 | Female | 1.6084 | 1.3509 | 1.8666 |
| DALYs | age_45-49 | Female | 1.4319 | 1.2265 | 1.6376 |
| DALYs | age_50-54 | Female | 1.4647 | 1.2924 | 1.6372 |
| DALYs | age_55-59 | Female | 1.458 | 1.3053 | 1.6109 |
| DALYs | age_60-64 | Female | 1.515 | 1.3798 | 1.6504 |
| DALYs | age_65-69 | Female | 1.521 | 1.3992 | 1.643 |
| DALYs | age_70-75 | Female | 1.519 | 1.4013 | 1.6369 |
| DALYs | age_75-79 | Female | 1.5592 | 1.4352 | 1.6832 |
| DALYs | age_80-84 | Female | 1.7119 | 1.5625 | 1.8615 |
| DALYs | age_85-89 | Female | 1.8651 | 1.6413 | 2.0894 |
| DALYs | age_90-94 | Female | 2.0877 | 1.6522 | 2.5252 |
| DALYs | age_95+ | Female | 2.1506 | 1.0212 | 3.2926 |
| DALYs | age_<5 | Male | -3.7024 | -4.0426 | -3.3611 |
| DALYs | age_5-9 | Male | -2.9689 | -3.5272 | -2.4074 |
| DALYs | age_10-14 | Male | -1.7506 | -2.5313 | -0.9635 |
| DALYs | age_15-19 | Male | 0.4238 | -0.4507 | 1.306 |
| DALYs | age_20-24 | Male | 2.5105 | 1.6987 | 3.3288 |
| DALYs | age_25-29 | Male | 3.4028 | 2.8058 | 4.0032 |
| DALYs | age_30-34 | Male | 3.374 | 2.9559 | 3.7938 |
| DALYs | age_35-39 | Male | 2.757 | 2.423 | 3.0922 |
| DALYs | age_40-44 | Male | 2.1788 | 1.914 | 2.4443 |
| DALYs | age_45-49 | Male | 1.8758 | 1.661 | 2.0911 |
| DALYs | age_50-54 | Male | 1.8481 | 1.6643 | 2.0323 |
| DALYs | age_55-59 | Male | 1.599 | 1.4331 | 1.7652 |
| DALYs | age_60-64 | Male | 1.4855 | 1.3357 | 1.6355 |
| DALYs | age_65-69 | Male | 1.3494 | 1.2095 | 1.4895 |
| DALYs | age_70-75 | Male | 1.2436 | 1.1015 | 1.3859 |
| DALYs | age_75-79 | Male | 1.1342 | 0.9733 | 1.2955 |
| DALYs | age_80-84 | Male | 1.3672 | 1.1528 | 1.5821 |
| DALYs | age_85-89 | Male | 1.7648 | 1.418 | 2.1129 |
| DALYs | age_90-94 | Male | 2.0689 | 1.3114 | 2.8321 |
| DALYs | age_95+ | Male | 2.7934 | -0.5257 | 6.2231 |
