# Supplementary material for: Trends and projections of PM2.5-attributable disease burden in China: a GBD 2021-based analysis
Source: Front Public Health. 2026 Jan 15;14:1684344. doi: 10.3389/fpubh.2026.1684344 (PMC12852448; doi:10.3389/fpubh.2026.1684344)
Supplement: Supplementary file 12 [file Table_4.DOCX]

| **Table S4. Annual percentage change in HAP-SF-attributable mortality and DALYs by age group and sex, 1990-2021 (local drift)** | | | | | |
| --- | --- | --- | --- | --- | --- |
| **Measure** | **Age** | **Sex** | **Percent per Year** | **95%CI_Low** | **95%CI_High** |
| Mortality | age_<5 | Both | -13.4683 | -14.0393 | -12.8935 |
| Mortality | age_5-9 | Both | -13.0635 | -13.7325 | -12.3892 |
| Mortality | age_10-14 | Both | -12.5387 | -13.4864 | -11.5806 |
| Mortality | age_15-19 | Both | -11.4472 | -12.5548 | -10.3256 |
| Mortality | age_20-24 | Both | -10.2487 | -11.3327 | -9.1515 |
| Mortality | age_25-29 | Both | -9.4818 | -10.3542 | -8.601 |
| Mortality | age_30-34 | Both | -8.6895 | -9.3203 | -8.0543 |
| Mortality | age_35-39 | Both | -8.6678 | -9.1409 | -8.1922 |
| Mortality | age_40-44 | Both | -8.8325 | -9.1662 | -8.4976 |
| Mortality | age_45-49 | Both | -9.0895 | -9.3272 | -8.8513 |
| Mortality | age_50-54 | Both | -9.3422 | -9.5235 | -9.1605 |
| Mortality | age_55-59 | Both | -9.6153 | -9.7662 | -9.4641 |
| Mortality | age_60-64 | Both | -9.4957 | -9.6175 | -9.3737 |
| Mortality | age_65-69 | Both | -9.3506 | -9.4504 | -9.2508 |
| Mortality | age_70-75 | Both | -9.1632 | -9.2508 | -9.0755 |
| Mortality | age_75-79 | Both | -9.0955 | -9.1771 | -9.0138 |
| Mortality | age_80-84 | Both | -9.0167 | -9.1014 | -8.932 |
| Mortality | age_85-89 | Both | -8.8766 | -8.9804 | -8.7727 |
| Mortality | age_90-94 | Both | -8.6955 | -8.8627 | -8.528 |
| Mortality | age_95+ | Both | -8.5672 | -8.9962 | -8.1361 |
| Mortality | age_<5 | Female | -13.7588 | -14.3665 | -13.1468 |
| Mortality | age_5-9 | Female | -13.2377 | -13.9595 | -12.5099 |
| Mortality | age_10-14 | Female | -12.7696 | -13.805 | -11.7217 |
| Mortality | age_15-19 | Female | -11.82 | -13.0416 | -10.5812 |
| Mortality | age_20-24 | Female | -10.8331 | -12.0391 | -9.6106 |
| Mortality | age_25-29 | Female | -10.2507 | -11.2354 | -9.255 |
| Mortality | age_30-34 | Female | -9.6037 | -10.3251 | -8.8766 |
| Mortality | age_35-39 | Female | -9.5248 | -10.0639 | -8.9824 |
| Mortality | age_40-44 | Female | -9.6449 | -10.0234 | -9.2649 |
| Mortality | age_45-49 | Female | -9.7981 | -10.0641 | -9.5314 |
| Mortality | age_50-54 | Female | -9.9398 | -10.1391 | -9.74 |
| Mortality | age_55-59 | Female | -10.0531 | -10.2165 | -9.8894 |
| Mortality | age_60-64 | Female | -9.785 | -9.9149 | -9.6549 |
| Mortality | age_65-69 | Female | -9.5116 | -9.6154 | -9.4076 |
| Mortality | age_70-75 | Female | -9.2643 | -9.3534 | -9.1752 |
| Mortality | age_75-79 | Female | -9.1433 | -9.2236 | -9.063 |
| Mortality | age_80-84 | Female | -9.0881 | -9.1682 | -9.0078 |
| Mortality | age_85-89 | Female | -9.0563 | -9.1518 | -8.9606 |
| Mortality | age_90-94 | Female | -8.8654 | -9.0114 | -8.7192 |
| Mortality | age_95+ | Female | -8.6856 | -9.009 | -8.361 |
| Mortality | age_<5 | Male | -13.2551 | -13.9605 | -12.5439 |
| Mortality | age_5-9 | Male | -12.9698 | -13.7886 | -12.1433 |
| Mortality | age_10-14 | Male | -12.4201 | -13.5706 | -11.2543 |
| Mortality | age_15-19 | Male | -11.2348 | -12.5719 | -9.8771 |
| Mortality | age_20-24 | Male | -9.8919 | -11.1946 | -8.5701 |
| Mortality | age_25-29 | Male | -9.0124 | -10.0524 | -7.9605 |
| Mortality | age_30-34 | Male | -8.1258 | -8.8724 | -7.373 |
| Mortality | age_35-39 | Male | -8.1442 | -8.7052 | -7.5797 |
| Mortality | age_40-44 | Male | -8.3306 | -8.7274 | -7.9321 |
| Mortality | age_45-49 | Male | -8.6313 | -8.9162 | -8.3454 |
| Mortality | age_50-54 | Male | -8.9257 | -9.1454 | -8.7053 |
| Mortality | age_55-59 | Male | -9.2878 | -9.4725 | -9.1026 |
| Mortality | age_60-64 | Male | -9.2721 | -9.4229 | -9.1211 |
| Mortality | age_65-69 | Male | -9.2283 | -9.354 | -9.1024 |
| Mortality | age_70-75 | Male | -9.1159 | -9.2285 | -9.0032 |
| Mortality | age_75-79 | Male | -9.1257 | -9.2337 | -9.0176 |
| Mortality | age_80-84 | Male | -9.0183 | -9.1347 | -8.9017 |
| Mortality | age_85-89 | Male | -8.7111 | -8.8593 | -8.5627 |
| Mortality | age_90-94 | Male | -8.4263 | -8.6845 | -8.1675 |
| Mortality | age_95+ | Male | -8.2269 | -9.2624 | -7.1797 |
| DALYs | age_<5 | Both | -13.4532 | -13.7855 | -13.1197 |
| DALYs | age_5-9 | Both | -12.9482 | -13.3527 | -12.5418 |
| DALYs | age_10-14 | Both | -12.2967 | -12.8767 | -11.7129 |
| DALYs | age_15-19 | Both | -11.0898 | -11.7687 | -10.4057 |
| DALYs | age_20-24 | Both | -9.8153 | -10.4795 | -9.1461 |
| DALYs | age_25-29 | Both | -8.9721 | -9.5085 | -8.4325 |
| DALYs | age_30-34 | Both | -8.2056 | -8.6021 | -7.8074 |
| DALYs | age_35-39 | Both | -8.2184 | -8.53 | -7.9058 |
| DALYs | age_40-44 | Both | -8.4277 | -8.6618 | -8.1931 |
| DALYs | age_45-49 | Both | -8.7201 | -8.8996 | -8.5402 |
| DALYs | age_50-54 | Both | -8.9986 | -9.1465 | -8.8504 |
| DALYs | age_55-59 | Both | -9.2531 | -9.3844 | -9.1217 |
| DALYs | age_60-64 | Both | -9.1756 | -9.2907 | -9.0603 |
| DALYs | age_65-69 | Both | -9.0441 | -9.1482 | -8.9399 |
| DALYs | age_70-75 | Both | -8.884 | -8.9853 | -8.7825 |
| DALYs | age_75-79 | Both | -8.824 | -8.9294 | -8.7185 |
| DALYs | age_80-84 | Both | -8.7604 | -8.8824 | -8.6381 |
| DALYs | age_85-89 | Both | -8.6302 | -8.7961 | -8.464 |
| DALYs | age_90-94 | Both | -8.4782 | -8.7741 | -8.1813 |
| DALYs | age_95+ | Both | -8.3821 | -9.1862 | -7.571 |
| DALYs | age_<5 | Female | -13.7407 | -14.055 | -13.4253 |
| DALYs | age_5-9 | Female | -13.0803 | -13.4631 | -12.6959 |
| DALYs | age_10-14 | Female | -12.3832 | -12.9331 | -11.8298 |
| DALYs | age_15-19 | Female | -11.2004 | -11.8453 | -10.5507 |
| DALYs | age_20-24 | Female | -10.017 | -10.6482 | -9.3814 |
| DALYs | age_25-29 | Female | -9.2821 | -9.7922 | -8.7691 |
| DALYs | age_30-34 | Female | -8.6555 | -9.0329 | -8.2766 |
| DALYs | age_35-39 | Female | -8.6826 | -8.9801 | -8.3841 |
| DALYs | age_40-44 | Female | -8.9172 | -9.1415 | -8.6924 |
| DALYs | age_45-49 | Female | -9.1702 | -9.3416 | -8.9984 |
| DALYs | age_50-54 | Female | -9.3851 | -9.525 | -9.245 |
| DALYs | age_55-59 | Female | -9.5151 | -9.6378 | -9.3922 |
| DALYs | age_60-64 | Female | -9.3126 | -9.419 | -9.2061 |
| DALYs | age_65-69 | Female | -9.0704 | -9.1647 | -8.9761 |
| DALYs | age_70-75 | Female | -8.8679 | -8.9577 | -8.778 |
| DALYs | age_75-79 | Female | -8.7755 | -8.8665 | -8.6845 |
| DALYs | age_80-84 | Female | -8.7396 | -8.8415 | -8.6375 |
| DALYs | age_85-89 | Female | -8.7244 | -8.8587 | -8.5899 |
| DALYs | age_90-94 | Female | -8.5719 | -8.7993 | -8.344 |
| DALYs | age_95+ | Female | -8.443 | -8.9794 | -7.9035 |
| DALYs | age_<5 | Male | -13.2424 | -13.6299 | -12.8533 |
| DALYs | age_5-9 | Male | -12.8864 | -13.3579 | -12.4124 |
| DALYs | age_10-14 | Male | -12.2732 | -12.948 | -11.5932 |
| DALYs | age_15-19 | Male | -11.0351 | -11.824 | -10.2391 |
| DALYs | age_20-24 | Male | -9.6734 | -10.4454 | -8.8948 |
| DALYs | age_25-29 | Male | -8.7415 | -9.3649 | -8.1138 |
| DALYs | age_30-34 | Male | -7.8687 | -8.3293 | -7.4058 |
| DALYs | age_35-39 | Male | -7.8811 | -8.2424 | -7.5185 |
| DALYs | age_40-44 | Male | -8.0831 | -8.3538 | -7.8115 |
| DALYs | age_45-49 | Male | -8.3979 | -8.6062 | -8.1891 |
| DALYs | age_50-54 | Male | -8.7055 | -8.8784 | -8.5324 |
| DALYs | age_55-59 | Male | -9.0406 | -9.1951 | -8.8858 |
| DALYs | age_60-64 | Male | -9.0603 | -9.1972 | -8.9232 |
| DALYs | age_65-69 | Male | -9.0255 | -9.1513 | -8.8996 |
| DALYs | age_70-75 | Male | -8.9288 | -9.0535 | -8.8039 |
| DALYs | age_75-79 | Male | -8.9271 | -9.0602 | -8.7938 |
| DALYs | age_80-84 | Male | -8.836 | -8.9959 | -8.6759 |
| DALYs | age_85-89 | Male | -8.5396 | -8.7656 | -8.3131 |
| DALYs | age_90-94 | Male | -8.2895 | -8.7258 | -7.8512 |
| DALYs | age_95+ | Male | -8.1167 | -9.9592 | -6.2366 |
