# Supplementary material for: Trends and projections of PM2.5-attributable disease burden in China: a GBD 2021-based analysis
Source: Front Public Health. 2026 Jan 15;14:1684344. doi: 10.3389/fpubh.2026.1684344 (PMC12852448; doi:10.3389/fpubh.2026.1684344)
Supplement: Supplementary file 13 [file Table_5.DOCX]

| **Table S5. Fitted longitudinal age effects of PMP rates (per 100 000 person-years) and the corresponding 95% CIs** | | | | | | |
| --- | --- | --- | --- | --- | --- | --- |
| **Measure** | **Age** | **Sex** | **Rate** | **95%CI_Low** | **95%CI_High** |  |
| Mortality | age_<5 | Both | 654.604 | 512.5054 | 836.1012 |  |
| Mortality | age_5-9 | Both | 8.65 | 6.6537 | 11.2452 |  |
| Mortality | age_10-14 | Both | 2.6212 | 1.9106 | 3.5961 |  |
| Mortality | age_15-19 | Both | 1.7051 | 1.2284 | 2.3667 |  |
| Mortality | age_20-24 | Both | 1.226 | 0.878 | 1.7119 |  |
| Mortality | age_25-29 | Both | 9.1164 | 8.0706 | 10.2979 |  |
| Mortality | age_30-34 | Both | 14.2486 | 13.0113 | 15.6037 |  |
| Mortality | age_35-39 | Both | 23.5689 | 22.0561 | 25.1854 |  |
| Mortality | age_40-44 | Both | 40.3997 | 38.4219 | 42.4792 |  |
| Mortality | age_45-49 | Both | 55.0294 | 52.7263 | 57.4332 |  |
| Mortality | age_50-54 | Both | 88.5034 | 85.353 | 91.7701 |  |
| Mortality | age_55-59 | Both | 126.6584 | 122.473 | 130.9869 |  |
| Mortality | age_60-64 | Both | 185.1151 | 179.2097 | 191.2151 |  |
| Mortality | age_65-69 | Both | 270.7395 | 261.4071 | 280.4052 |  |
| Mortality | age_70-75 | Both | 441.2796 | 426.0736 | 457.0283 |  |
| Mortality | age_75-79 | Both | 633.3401 | 611.2455 | 656.2334 |  |
| Mortality | age_80-84 | Both | 959.9959 | 925.8948 | 995.3531 |  |
| Mortality | age_85-89 | Both | 1579.72 | 1521.683 | 1639.971 |  |
| Mortality | age_90-94 | Both | 2058.266 | 1974.058 | 2146.066 |  |
| Mortality | age_95+ | Both | 1965.626 | 1846.632 | 2092.286 |  |
| Mortality | age_<5 | Female | 1054.991 | 805.292 | 1382.114 |  |
| Mortality | age_5-9 | Female | 13.0658 | 9.8829 | 17.2739 |  |
| Mortality | age_10-14 | Female | 3.7978 | 2.7575 | 5.2306 |  |
| Mortality | age_15-19 | Female | 1.9916 | 1.4106 | 2.8121 |  |
| Mortality | age_20-24 | Female | 1.465 | 1.0514 | 2.0414 |  |
| Mortality | age_25-29 | Female | 8.8414 | 7.7642 | 10.068 |  |
| Mortality | age_30-34 | Female | 12.0379 | 10.903 | 13.2908 |  |
| Mortality | age_35-39 | Female | 18.4352 | 17.1509 | 19.8156 |  |
| Mortality | age_40-44 | Female | 29.6025 | 28.0305 | 31.2626 |  |
| Mortality | age_45-49 | Female | 38.5879 | 36.8376 | 40.4213 |  |
| Mortality | age_50-54 | Female | 62.794 | 60.3843 | 65.2999 |  |
| Mortality | age_55-59 | Female | 86.2276 | 83.1393 | 89.4306 |  |
| Mortality | age_60-64 | Female | 123.2736 | 119.0449 | 127.6525 |  |
| Mortality | age_65-69 | Female | 179.7764 | 173.1697 | 186.6351 |  |
| Mortality | age_70-75 | Female | 291.9991 | 281.3084 | 303.096 |  |
| Mortality | age_75-79 | Female | 421.3495 | 405.8074 | 437.487 |  |
| Mortality | age_80-84 | Female | 646.3095 | 622.229 | 671.3218 |  |
| Mortality | age_85-89 | Female | 908.1231 | 873.3566 | 944.2737 |  |
| Mortality | age_90-94 | Female | 1176.147 | 1127.89 | 1226.469 |  |
| Mortality | age_95+ | Female | 1444.298 | 1370.479 | 1522.092 |  |
| Mortality | age_<5 | Male | 527.7645 | 386.7235 | 720.2443 |  |
| Mortality | age_5-9 | Male | 7.299 | 5.186 | 10.2731 |  |
| Mortality | age_10-14 | Male | 2.2645 | 1.4831 | 3.4577 |  |
| Mortality | age_15-19 | Male | 1.6881 | 1.1028 | 2.5841 |  |
| Mortality | age_20-24 | Male | 1.1824 | 0.7543 | 1.8535 |  |
| Mortality | age_25-29 | Male | 9.8625 | 8.4283 | 11.5409 |  |
| Mortality | age_30-34 | Male | 16.5008 | 14.6986 | 18.5239 |  |
| Mortality | age_35-39 | Male | 28.3616 | 26.0645 | 30.8613 |  |
| Mortality | age_40-44 | Male | 50.3106 | 47.1945 | 53.6324 |  |
| Mortality | age_45-49 | Male | 70.3019 | 66.5734 | 74.2392 |  |
| Mortality | age_50-54 | Male | 112.9161 | 107.8035 | 118.2711 |  |
| Mortality | age_55-59 | Male | 165.9688 | 159.0104 | 173.2317 |  |
| Mortality | age_60-64 | Male | 246.9352 | 236.8919 | 257.4043 |  |
| Mortality | age_65-69 | Male | 364.6791 | 348.5718 | 381.5307 |  |
| Mortality | age_70-75 | Male | 601.2477 | 574.6301 | 629.0983 |  |
| Mortality | age_75-79 | Male | 871.4068 | 832.1931 | 912.4683 |  |
| Mortality | age_80-84 | Male | 1340.781 | 1278.825 | 1405.738 |  |
| Mortality | age_85-89 | Male | 2749.682 | 2617.683 | 2888.337 |  |
| Mortality | age_90-94 | Male | 4168.93 | 3936.109 | 4415.522 |  |
| Mortality | age_95+ | Male | 2643.899 | 2329.238 | 3001.068 |  |
| DALYs | age_<5 | Both | 47996.12 | 41121.47 | 56020.06 |  |
| DALYs | age_5-9 | Both | 607.8532 | 514.5539 | 718.0697 |  |
| DALYs | age_10-14 | Both | 176.7912 | 143.9153 | 217.1774 |  |
| DALYs | age_15-19 | Both | 110.3815 | 88.5652 | 137.5718 |  |
| DALYs | age_20-24 | Both | 76.3042 | 60.5613 | 96.1396 |  |
| DALYs | age_25-29 | Both | 702.2278 | 647.4546 | 761.6346 |  |
| DALYs | age_30-34 | Both | 968.9544 | 908.1584 | 1033.82 |  |
| DALYs | age_35-39 | Both | 1411.789 | 1342.58 | 1484.565 |  |
| DALYs | age_40-44 | Both | 2140.465 | 2055.343 | 2229.111 |  |
| DALYs | age_45-49 | Both | 2668.797 | 2574.114 | 2766.962 |  |
| DALYs | age_50-54 | Both | 3814.439 | 3693.642 | 3939.187 |  |
| DALYs | age_55-59 | Both | 4819.199 | 4672.21 | 4970.812 |  |
| DALYs | age_60-64 | Both | 6080.738 | 5896.595 | 6270.632 |  |
| DALYs | age_65-69 | Both | 7534.573 | 7284.295 | 7793.449 |  |
| DALYs | age_70-75 | Both | 10068.98 | 9728.099 | 10421.81 |  |
| DALYs | age_75-79 | Both | 11612.89 | 11203.9 | 12036.8 |  |
| DALYs | age_80-84 | Both | 13724.87 | 13211.96 | 14257.68 |  |
| DALYs | age_85-89 | Both | 17688.15 | 16952.11 | 18456.15 |  |
| DALYs | age_90-94 | Both | 19977.45 | 18891.35 | 21125.99 |  |
| DALYs | age_95+ | Both | 18180.63 | 16328.98 | 20242.24 |  |
| DALYs | age_<5 | Female | 63448.83 | 54997.59 | 73198.75 |  |
| DALYs | age_5-9 | Female | 762.4851 | 655.4715 | 886.97 |  |
| DALYs | age_10-14 | Female | 217.2673 | 181.2018 | 260.5109 |  |
| DALYs | age_15-19 | Female | 112.9295 | 92.1504 | 138.394 |  |
| DALYs | age_20-24 | Female | 82.4704 | 67.3697 | 100.9558 |  |
| DALYs | age_25-29 | Female | 713.2722 | 663.0349 | 767.316 |  |
| DALYs | age_30-34 | Female | 871.6614 | 821.1802 | 925.2459 |  |
| DALYs | age_35-39 | Female | 1165.31 | 1112.166 | 1220.993 |  |
| DALYs | age_40-44 | Female | 1642.702 | 1581.471 | 1706.303 |  |
| DALYs | age_45-49 | Female | 1975.782 | 1910.029 | 2043.798 |  |
| DALYs | age_50-54 | Female | 2843.069 | 2758.888 | 2929.818 |  |
| DALYs | age_55-59 | Female | 3474.696 | 3375.391 | 3576.922 |  |
| DALYs | age_60-64 | Female | 4301.315 | 4180.303 | 4425.829 |  |
| DALYs | age_65-69 | Female | 5318.025 | 5155.258 | 5485.93 |  |
| DALYs | age_70-75 | Female | 7066.687 | 6847.483 | 7292.908 |  |
| DALYs | age_75-79 | Female | 8169.106 | 7907.932 | 8438.906 |  |
| DALYs | age_80-84 | Female | 9768.089 | 9442.795 | 10104.59 |  |
| DALYs | age_85-89 | Female | 10879.23 | 10482.01 | 11291.49 |  |
| DALYs | age_90-94 | Female | 12171.3 | 11628.45 | 12739.49 |  |
| DALYs | age_95+ | Female | 14107.06 | 13129.77 | 15157.09 |  |
| DALYs | age_<5 | Male | 41385.1 | 34486.17 | 49664.16 |  |
| DALYs | age_5-9 | Male | 543.8373 | 445.6739 | 663.6219 |  |
| DALYs | age_10-14 | Male | 160.5779 | 125.0292 | 206.2339 |  |
| DALYs | age_15-19 | Male | 113.6514 | 87.6769 | 147.3211 |  |
| DALYs | age_20-24 | Male | 75.764 | 57.1454 | 100.4487 |  |
| DALYs | age_25-29 | Male | 722.794 | 655.6239 | 796.8459 |  |
| DALYs | age_30-34 | Male | 1072.358 | 993.3166 | 1157.688 |  |
| DALYs | age_35-39 | Male | 1642.109 | 1547.953 | 1741.992 |  |
| DALYs | age_40-44 | Male | 2597.06 | 2476.724 | 2723.243 |  |
| DALYs | age_45-49 | Male | 3313.415 | 3176.48 | 3456.254 |  |
| DALYs | age_50-54 | Male | 4737.863 | 4562.738 | 4919.71 |  |
| DALYs | age_55-59 | Male | 6125.585 | 5907.779 | 6351.42 |  |
| DALYs | age_60-64 | Male | 7853.703 | 7574.973 | 8142.688 |  |
| DALYs | age_65-69 | Male | 9811.154 | 9427.196 | 10210.75 |  |
| DALYs | age_70-75 | Male | 13271.73 | 12740.18 | 13825.44 |  |
| DALYs | age_75-79 | Male | 15483.09 | 14833.17 | 16161.49 |  |
| DALYs | age_80-84 | Male | 18557.78 | 17715.96 | 19439.6 |  |
| DALYs | age_85-89 | Male | 29497.59 | 27990.9 | 31085.38 |  |
| DALYs | age_90-94 | Male | 38752.28 | 35975.15 | 41743.78 |  |
| DALYs | age_95+ | Male | 23515.75 | 18955.13 | 29173.67 |  |
