# Supplementary material for: Trends and projections of PM2.5-attributable disease burden in China: a GBD 2021-based analysis
Source: Front Public Health. 2026 Jan 15;14:1684344. doi: 10.3389/fpubh.2026.1684344 (PMC12852448; doi:10.3389/fpubh.2026.1684344)
Supplement: Supplementary file 14 [file Table_6.DOCX]

| **Table S6. Fitted longitudinal age effects of APMP rates (per 100 000 person-years) and the corresponding 95% CIs** | | | | | |
| --- | --- | --- | --- | --- | --- |
| **Measure** | **Age** | **Sex** | **Rate** | **95%CI_Low** | **95%CI_High** |
| Mortality | age_<5 | Both | 15.6367 | 11.8403 | 20.6503 |
| Mortality | age_5-9 | Both | 0.2808 | 0.1951 | 0.4042 |
| Mortality | age_10-14 | Both | 0.1184 | 0.075 | 0.1868 |
| Mortality | age_15-19 | Both | 0.0984 | 0.0615 | 0.1573 |
| Mortality | age_20-24 | Both | 0.0952 | 0.0599 | 0.1511 |
| Mortality | age_25-29 | Both | 1.1929 | 1.0187 | 1.3968 |
| Mortality | age_30-34 | Both | 2.4869 | 2.2171 | 2.7894 |
| Mortality | age_35-39 | Both | 5.4558 | 5.0203 | 5.929 |
| Mortality | age_40-44 | Both | 12.4383 | 11.6987 | 13.2246 |
| Mortality | age_45-49 | Both | 22.5249 | 21.4302 | 23.6754 |
| Mortality | age_50-54 | Both | 45.1463 | 43.373 | 46.992 |
| Mortality | age_55-59 | Both | 84.361 | 81.4953 | 87.3275 |
| Mortality | age_60-64 | Both | 162.4116 | 157.2233 | 167.7711 |
| Mortality | age_65-69 | Both | 308.9851 | 297.6039 | 320.8015 |
| Mortality | age_70-75 | Both | 660.1106 | 635.4266 | 685.7536 |
| Mortality | age_75-79 | Both | 1256.997 | 1208.858 | 1307.053 |
| Mortality | age_80-84 | Both | 2514.699 | 2415.76 | 2617.69 |
| Mortality | age_85-89 | Both | 5393.482 | 5172.997 | 5623.366 |
| Mortality | age_90-94 | Both | 9085.035 | 8676.044 | 9513.306 |
| Mortality | age_95+ | Both | 11081.36 | 10383.69 | 11825.9 |
| Mortality | age_<5 | Female | 19.0792 | 13.7517 | 26.4704 |
| Mortality | age_5-9 | Female | 0.3457 | 0.2329 | 0.5132 |
| Mortality | age_10-14 | Female | 0.1417 | 0.0882 | 0.2278 |
| Mortality | age_15-19 | Female | 0.0998 | 0.0602 | 0.1655 |
| Mortality | age_20-24 | Female | 0.1 | 0.0622 | 0.1609 |
| Mortality | age_25-29 | Female | 0.8683 | 0.7193 | 1.0481 |
| Mortality | age_30-34 | Female | 1.5868 | 1.3792 | 1.8255 |
| Mortality | age_35-39 | Female | 3.3324 | 3.0115 | 3.6876 |
| Mortality | age_40-44 | Female | 7.4229 | 6.8926 | 7.994 |
| Mortality | age_45-49 | Female | 13.4292 | 12.6522 | 14.254 |
| Mortality | age_50-54 | Female | 28.329 | 27.025 | 29.696 |
| Mortality | age_55-59 | Female | 53.1185 | 51.0168 | 55.3068 |
| Mortality | age_60-64 | Female | 104.5368 | 100.7297 | 108.4878 |
| Mortality | age_65-69 | Female | 207.2917 | 198.5962 | 216.3679 |
| Mortality | age_70-75 | Female | 460.9962 | 441.4492 | 481.4088 |
| Mortality | age_75-79 | Female | 913.7094 | 874.2358 | 954.9654 |
| Mortality | age_80-84 | Female | 1940.332 | 1854.947 | 2029.648 |
| Mortality | age_85-89 | Female | 3634.201 | 3468.971 | 3807.301 |
| Mortality | age_90-94 | Female | 6357.39 | 6049.771 | 6680.651 |
| Mortality | age_95+ | Female | 10280.21 | 9687.19 | 10909.53 |
| Mortality | age_<5 | Male | 15.1915 | 10.9247 | 21.1248 |
| Mortality | age_5-9 | Male | 0.2702 | 0.1718 | 0.4249 |
| Mortality | age_10-14 | Male | 0.1153 | 0.0646 | 0.2058 |
| Mortality | age_15-19 | Male | 0.1059 | 0.0593 | 0.1894 |
| Mortality | age_20-24 | Male | 0.0996 | 0.0553 | 0.1793 |
| Mortality | age_25-29 | Male | 1.5057 | 1.2507 | 1.8127 |
| Mortality | age_30-34 | Male | 3.3043 | 2.8908 | 3.7768 |
| Mortality | age_35-39 | Male | 7.3794 | 6.6977 | 8.1304 |
| Mortality | age_40-44 | Male | 17.0401 | 15.8625 | 18.3052 |
| Mortality | age_45-49 | Male | 31.0091 | 29.2488 | 32.8754 |
| Mortality | age_50-54 | Male | 61.2297 | 58.3992 | 64.1973 |
| Mortality | age_55-59 | Male | 114.7956 | 110.1903 | 119.5935 |
| Mortality | age_60-64 | Male | 220.1533 | 211.7614 | 228.8777 |
| Mortality | age_65-69 | Male | 414.3994 | 396.178 | 433.4588 |
| Mortality | age_70-75 | Male | 876.9224 | 837.6783 | 918.0051 |
| Mortality | age_75-79 | Male | 1661.225 | 1584.83 | 1741.303 |
| Mortality | age_80-84 | Male | 3299.795 | 3142.755 | 3464.681 |
| Mortality | age_85-89 | Male | 8569.621 | 8144.626 | 9016.793 |
| Mortality | age_90-94 | Male | 16295.51 | 15373.97 | 17272.29 |
| Mortality | age_95+ | Male | 13981.49 | 12472.38 | 15673.2 |
| DALYs | age_<5 | Both | 1140.547 | 977.6694 | 1330.56 |
| DALYs | age_5-9 | Both | 19.755 | 16.1381 | 24.1825 |
| DALYs | age_10-14 | Both | 7.9511 | 6.1305 | 10.3124 |
| DALYs | age_15-19 | Both | 6.3123 | 4.7893 | 8.3196 |
| DALYs | age_20-24 | Both | 5.7954 | 4.3752 | 7.6766 |
| DALYs | age_25-29 | Both | 92.5771 | 84.4373 | 101.5016 |
| DALYs | age_30-34 | Both | 169.6923 | 157.9449 | 182.3135 |
| DALYs | age_35-39 | Both | 326.892 | 309.2008 | 345.5954 |
| DALYs | age_40-44 | Both | 656.7775 | 628.6737 | 686.1376 |
| DALYs | age_45-49 | Both | 1085.692 | 1046.107 | 1126.776 |
| DALYs | age_50-54 | Both | 1941.366 | 1881.321 | 2003.327 |
| DALYs | age_55-59 | Both | 3197.093 | 3107.558 | 3289.207 |
| DALYs | age_60-64 | Both | 5300.111 | 5154.982 | 5449.326 |
| DALYs | age_65-69 | Both | 8522.71 | 8249.437 | 8805.035 |
| DALYs | age_70-75 | Both | 14882.49 | 14388.25 | 15393.7 |
| DALYs | age_75-79 | Both | 22699.55 | 21908.07 | 23519.63 |
| DALYs | age_80-84 | Both | 35298.03 | 33989.49 | 36656.93 |
| DALYs | age_85-89 | Both | 59185.28 | 56766.13 | 61707.53 |
| DALYs | age_90-94 | Both | 86495.14 | 82037.05 | 91195.5 |
| DALYs | age_95+ | Both | 100478.8 | 91320.9 | 110555 |
| DALYs | age_<5 | Female | 1132.13 | 966.9203 | 1325.568 |
| DALYs | age_5-9 | Female | 20.1301 | 16.5146 | 24.5372 |
| DALYs | age_10-14 | Female | 8.0351 | 6.2699 | 10.2973 |
| DALYs | age_15-19 | Female | 5.5611 | 4.2293 | 7.3124 |
| DALYs | age_20-24 | Female | 5.406 | 4.1391 | 7.0606 |
| DALYs | age_25-29 | Female | 72.3865 | 65.7954 | 79.638 |
| DALYs | age_30-34 | Female | 118.1451 | 109.4539 | 127.5265 |
| DALYs | age_35-39 | Female | 214.3043 | 201.8223 | 227.5583 |
| DALYs | age_40-44 | Female | 414.2935 | 395.1011 | 434.4182 |
| DALYs | age_45-49 | Female | 687.1107 | 660.1385 | 715.185 |
| DALYs | age_50-54 | Female | 1283.934 | 1241.656 | 1327.652 |
| DALYs | age_55-59 | Female | 2133.439 | 2070.323 | 2198.48 |
| DALYs | age_60-64 | Female | 3612.913 | 3510.367 | 3718.455 |
| DALYs | age_65-69 | Female | 6032.186 | 5831.522 | 6239.756 |
| DALYs | age_70-75 | Female | 10899.54 | 10525.66 | 11286.71 |
| DALYs | age_75-79 | Female | 17213.3 | 16598.17 | 17851.24 |
| DALYs | age_80-84 | Female | 28356.59 | 27297.74 | 29456.5 |
| DALYs | age_85-89 | Female | 42046.32 | 40339.39 | 43825.47 |
| DALYs | age_90-94 | Female | 63536.28 | 60495.28 | 66730.14 |
| DALYs | age_95+ | Female | 96915.41 | 90194.79 | 104136.8 |
| DALYs | age_<5 | Male | 1188.622 | 997.096 | 1416.937 |
| DALYs | age_5-9 | Male | 20.216 | 15.9483 | 25.6258 |
| DALYs | age_10-14 | Male | 8.177 | 5.9996 | 11.1445 |
| DALYs | age_15-19 | Male | 7.1095 | 5.1615 | 9.7928 |
| DALYs | age_20-24 | Male | 6.301 | 4.5068 | 8.8095 |
| DALYs | age_25-29 | Male | 111.7176 | 100.6633 | 123.9859 |
| DALYs | age_30-34 | Male | 216.6073 | 199.9335 | 234.6717 |
| DALYs | age_35-39 | Male | 429.1321 | 403.4305 | 456.471 |
| DALYs | age_40-44 | Male | 879.7627 | 838.1743 | 923.4146 |
| DALYs | age_45-49 | Male | 1457.946 | 1399.053 | 1519.319 |
| DALYs | age_50-54 | Male | 2569.252 | 2480.751 | 2660.911 |
| DALYs | age_55-59 | Male | 4231.137 | 4098.644 | 4367.913 |
| DALYs | age_60-64 | Male | 6978.861 | 6763.157 | 7201.445 |
| DALYs | age_65-69 | Male | 11098.73 | 10697.14 | 11515.39 |
| DALYs | age_70-75 | Male | 19225.72 | 18503.27 | 19976.36 |
| DALYs | age_75-79 | Male | 29226.94 | 28067.7 | 30434.06 |
| DALYs | age_80-84 | Male | 45107.39 | 43176.45 | 47124.7 |
| DALYs | age_85-89 | Male | 90679.45 | 86361.23 | 95213.59 |
| DALYs | age_90-94 | Male | 149531.7 | 139954.5 | 159764.3 |
| DALYs | age_95+ | Male | 122722.8 | 103213 | 145920.5 |
