# Supplementary material for: Trends and projections of PM2.5-attributable disease burden in China: a GBD 2021-based analysis
Source: Front Public Health. 2026 Jan 15;14:1684344. doi: 10.3389/fpubh.2026.1684344 (PMC12852448; doi:10.3389/fpubh.2026.1684344)
Supplement: Supplementary file 15 [file Table_7.DOCX]

| **Table S7. Fitted longitudinal age effects of HAP-SF rates (per 100 000 person-years) and the corresponding 95% CIs** | | | | | |
| --- | --- | --- | --- | --- | --- |
| **Measure** | **Age** | **Sex** | **Rate** | **95%CI_Low** | **95%CI_High** |
| Mortality | age_<5 | Both | 5374.404 | 4146.119 | 6966.567 |
| Mortality | age_5-9 | Both | 53.9896 | 41.978 | 69.4383 |
| Mortality | age_10-14 | Both | 11.981 | 9.1409 | 15.7035 |
| Mortality | age_15-19 | Both | 5.7763 | 4.4095 | 7.5667 |
| Mortality | age_20-24 | Both | 3.0451 | 2.3158 | 4.004 |
| Mortality | age_25-29 | Both | 14.8279 | 13.3112 | 16.5173 |
| Mortality | age_30-34 | Both | 17.434 | 16.067 | 18.9174 |
| Mortality | age_35-39 | Both | 21.6196 | 20.3817 | 22.9327 |
| Mortality | age_40-44 | Both | 27.5592 | 26.3325 | 28.843 |
| Mortality | age_45-49 | Both | 27.6171 | 26.5232 | 28.756 |
| Mortality | age_50-54 | Both | 34.473 | 33.2331 | 35.7592 |
| Mortality | age_55-59 | Both | 36.9746 | 35.6441 | 38.3547 |
| Mortality | age_60-64 | Both | 40.524 | 39.0578 | 42.0453 |
| Mortality | age_65-69 | Both | 45.0542 | 43.369 | 46.8049 |
| Mortality | age_70-75 | Both | 55.3426 | 53.2551 | 57.512 |
| Mortality | age_75-79 | Both | 58.7696 | 56.509 | 61.1207 |
| Mortality | age_80-84 | Both | 65.4804 | 62.893 | 68.1744 |
| Mortality | age_85-89 | Both | 79.816 | 76.5199 | 83.254 |
| Mortality | age_90-94 | Both | 77.6441 | 74.0554 | 81.4067 |
| Mortality | age_95+ | Both | 56.5789 | 52.7132 | 60.7281 |
| Mortality | age_<5 | Female | 6666.303 | 4987.741 | 8909.763 |
| Mortality | age_5-9 | Female | 62.83 | 47.4558 | 83.185 |
| Mortality | age_10-14 | Female | 13.7859 | 10.2867 | 18.4752 |
| Mortality | age_15-19 | Female | 5.5244 | 4.0769 | 7.486 |
| Mortality | age_20-24 | Female | 3.1128 | 2.3275 | 4.1629 |
| Mortality | age_25-29 | Female | 14.0434 | 12.4846 | 15.7968 |
| Mortality | age_30-34 | Female | 14.9755 | 13.6794 | 16.3944 |
| Mortality | age_35-39 | Female | 17.7747 | 16.6516 | 18.9735 |
| Mortality | age_40-44 | Female | 21.8641 | 20.7913 | 22.9923 |
| Mortality | age_45-49 | Female | 21.6032 | 20.6622 | 22.5871 |
| Mortality | age_50-54 | Female | 27.7369 | 26.6477 | 28.8707 |
| Mortality | age_55-59 | Female | 29.1667 | 28.023 | 30.3571 |
| Mortality | age_60-64 | Female | 31.9748 | 30.7219 | 33.2788 |
| Mortality | age_65-69 | Female | 36.0598 | 34.605 | 37.5758 |
| Mortality | age_70-75 | Female | 44.8246 | 43.0081 | 46.7177 |
| Mortality | age_75-79 | Female | 48.8444 | 46.8376 | 50.9372 |
| Mortality | age_80-84 | Female | 55.4064 | 53.0875 | 57.8267 |
| Mortality | age_85-89 | Female | 58.9047 | 56.3502 | 61.5751 |
| Mortality | age_90-94 | Female | 56.5954 | 53.9395 | 59.3822 |
| Mortality | age_95+ | Female | 52.9695 | 49.8259 | 56.3115 |
| Mortality | age_<5 | Male | 4788.758 | 3507.06 | 6538.869 |
| Mortality | age_5-9 | Male | 50.3751 | 37.213 | 68.1926 |
| Mortality | age_10-14 | Male | 11.2785 | 8.0904 | 15.7228 |
| Mortality | age_15-19 | Male | 6.1692 | 4.4696 | 8.515 |
| Mortality | age_20-24 | Male | 3.1156 | 2.2188 | 4.3747 |
| Mortality | age_25-29 | Male | 15.8675 | 13.9135 | 18.096 |
| Mortality | age_30-34 | Male | 19.7931 | 17.9395 | 21.8383 |
| Mortality | age_35-39 | Male | 25.1807 | 23.4536 | 27.0351 |
| Mortality | age_40-44 | Male | 32.8287 | 31.0743 | 34.6821 |
| Mortality | age_45-49 | Male | 33.2533 | 31.6684 | 34.9174 |
| Mortality | age_50-54 | Male | 40.8586 | 39.081 | 42.717 |
| Mortality | age_55-59 | Male | 44.4958 | 42.5565 | 46.5235 |
| Mortality | age_60-64 | Male | 48.869 | 46.7178 | 51.1193 |
| Mortality | age_65-69 | Male | 54.0018 | 51.5363 | 56.5852 |
| Mortality | age_70-75 | Male | 66.0751 | 63.0214 | 69.2768 |
| Mortality | age_75-79 | Male | 69.1303 | 65.8534 | 72.5702 |
| Mortality | age_80-84 | Male | 76.651 | 72.8817 | 80.6151 |
| Mortality | age_85-89 | Male | 115.3743 | 109.3997 | 121.6752 |
| Mortality | age_90-94 | Male | 129.7139 | 121.8471 | 138.0887 |
| Mortality | age_95+ | Male | 49.6581 | 42.6718 | 57.7884 |
| DALYs | age_<5 | Both | 399833.1 | 340963.2 | 468867.3 |
| DALYs | age_5-9 | Both | 3811.328 | 3265.552 | 4448.32 |
| DALYs | age_10-14 | Both | 808.2664 | 682.3733 | 957.3858 |
| DALYs | age_15-19 | Both | 373.4928 | 313.7402 | 444.6255 |
| DALYs | age_20-24 | Both | 190.3542 | 158.6871 | 228.3406 |
| DALYs | age_25-29 | Both | 1130.075 | 1053.625 | 1212.072 |
| DALYs | age_30-34 | Both | 1180.287 | 1115.494 | 1248.843 |
| DALYs | age_35-39 | Both | 1293.327 | 1239.059 | 1349.972 |
| DALYs | age_40-44 | Both | 1465.038 | 1414.462 | 1517.422 |
| DALYs | age_45-49 | Both | 1350.936 | 1307.762 | 1395.534 |
| DALYs | age_50-54 | Both | 1499.112 | 1453.729 | 1545.911 |
| DALYs | age_55-59 | Both | 1425.382 | 1380.939 | 1471.256 |
| DALYs | age_60-64 | Both | 1355.767 | 1312.242 | 1400.735 |
| DALYs | age_65-69 | Both | 1285.117 | 1241.571 | 1330.19 |
| DALYs | age_70-75 | Both | 1304.156 | 1258.466 | 1351.505 |
| DALYs | age_75-79 | Both | 1122.322 | 1080.871 | 1165.362 |
| DALYs | age_80-84 | Both | 982.991 | 943.9162 | 1023.684 |
| DALYs | age_85-89 | Both | 945.5802 | 903.2472 | 989.8972 |
| DALYs | age_90-94 | Both | 801.8037 | 755.0204 | 851.4859 |
| DALYs | age_95+ | Both | 561.1772 | 499.9571 | 629.8938 |
| DALYs | age_<5 | Female | 425088.5 | 365261.3 | 494715 |
| DALYs | age_5-9 | Female | 3835.331 | 3310.888 | 4442.845 |
| DALYs | age_10-14 | Female | 817.4531 | 697.1981 | 958.45 |
| DALYs | age_15-19 | Female | 321.9785 | 271.0585 | 382.4642 |
| DALYs | age_20-24 | Female | 180.3442 | 152.0962 | 213.8386 |
| DALYs | age_25-29 | Female | 1119.752 | 1049.234 | 1195.01 |
| DALYs | age_30-34 | Female | 1075.317 | 1019.441 | 1134.257 |
| DALYs | age_35-39 | Female | 1117.972 | 1073.277 | 1164.529 |
| DALYs | age_40-44 | Female | 1213.921 | 1173.761 | 1255.456 |
| DALYs | age_45-49 | Female | 1111.737 | 1077.743 | 1146.804 |
| DALYs | age_50-54 | Female | 1264.362 | 1227.997 | 1301.804 |
| DALYs | age_55-59 | Female | 1188.44 | 1153.37 | 1224.576 |
| DALYs | age_60-64 | Female | 1135.267 | 1101.021 | 1170.579 |
| DALYs | age_65-69 | Female | 1094.735 | 1060.079 | 1130.525 |
| DALYs | age_70-75 | Female | 1124.422 | 1087.88 | 1162.192 |
| DALYs | age_75-79 | Female | 991.6659 | 958.0381 | 1026.474 |
| DALYs | age_80-84 | Female | 885.6986 | 853.9064 | 918.6746 |
| DALYs | age_85-89 | Female | 752.2419 | 722.2933 | 783.4321 |
| DALYs | age_90-94 | Female | 629.0536 | 598.1697 | 661.5321 |
| DALYs | age_95+ | Female | 560.5239 | 517.1346 | 607.5537 |
| DALYs | age_<5 | Male | 386317.7 | 321093.2 | 464791.4 |
| DALYs | age_5-9 | Male | 3829.562 | 3200.116 | 4582.815 |
| DALYs | age_10-14 | Male | 811.0711 | 665.0714 | 989.1214 |
| DALYs | age_15-19 | Male | 419.3834 | 344.1335 | 511.0877 |
| DALYs | age_20-24 | Male | 201.7942 | 162.9526 | 249.8942 |
| DALYs | age_25-29 | Male | 1154.395 | 1062.494 | 1254.245 |
| DALYs | age_30-34 | Male | 1281.914 | 1200.141 | 1369.258 |
| DALYs | age_35-39 | Male | 1455.764 | 1385.068 | 1530.068 |
| DALYs | age_40-44 | Male | 1697.438 | 1629.85 | 1767.828 |
| DALYs | age_45-49 | Male | 1574.955 | 1516.829 | 1635.307 |
| DALYs | age_50-54 | Male | 1721.458 | 1660.977 | 1784.141 |
| DALYs | age_55-59 | Male | 1652.55 | 1592.577 | 1714.782 |
| DALYs | age_60-64 | Male | 1569.234 | 1510.274 | 1630.495 |
| DALYs | age_65-69 | Male | 1472.769 | 1414.073 | 1533.901 |
| DALYs | age_70-75 | Male | 1486.72 | 1425.064 | 1551.043 |
| DALYs | age_75-79 | Male | 1260.426 | 1204.779 | 1318.643 |
| DALYs | age_80-84 | Male | 1095.28 | 1042.245 | 1151.014 |
| DALYs | age_85-89 | Male | 1284.408 | 1213.394 | 1359.577 |
| DALYs | age_90-94 | Male | 1255.539 | 1158.323 | 1360.914 |
| DALYs | age_95+ | Male | 463.9798 | 358.0747 | 601.2076 |
