# Supplementary material for: Trends and projections of PM2.5-attributable disease burden in China: a GBD 2021-based analysis
Source: Front Public Health. 2026 Jan 15;14:1684344. doi: 10.3389/fpubh.2026.1684344 (PMC12852448; doi:10.3389/fpubh.2026.1684344)
Supplement: Supplementary file 16 [file Table_8.DOCX]

| **Table S8. Relative risk for PMP Mortality rate and DALYs rate of each period compared with the reference (2002–2006)** | | | | | |
| --- | --- | --- | --- | --- | --- |
| **Measure** | **Period** | **Sex** | **Rate Ratio** | **95%CI_Low** | **95%CI_High** |
| Mortality | period_1992 | Both | 1.3063 | 1.2708 | 1.3428 |
| Mortality | period_1997 | Both | 1.135 | 1.1129 | 1.1577 |
| Mortality | period_2002 | Both | 1 | 1 | 1 |
| Mortality | period_2007 | Both | 0.7633 | 0.7485 | 0.7784 |
| Mortality | period_2012 | Both | 0.6071 | 0.5907 | 0.624 |
| Mortality | period_2017 | Both | 0.4493 | 0.4331 | 0.4661 |
| Mortality | period_1992 | Female | 1.4553 | 1.4134 | 1.4985 |
| Mortality | period_1997 | Female | 1.2025 | 1.1789 | 1.2265 |
| Mortality | period_2002 | Female | 1 | 1 | 1 |
| Mortality | period_2007 | Female | 0.7167 | 0.7026 | 0.7311 |
| Mortality | period_2012 | Female | 0.5331 | 0.5177 | 0.549 |
| Mortality | period_2017 | Female | 0.3915 | 0.376 | 0.4076 |
| Mortality | period_1992 | Male | 1.2247 | 1.1811 | 1.2698 |
| Mortality | period_1997 | Male | 1.0929 | 1.064 | 1.1226 |
| Mortality | period_2002 | Male | 1 | 1 | 1 |
| Mortality | period_2007 | Male | 0.7926 | 0.7719 | 0.8138 |
| Mortality | period_2012 | Male | 0.6543 | 0.6314 | 0.6781 |
| Mortality | period_2017 | Male | 0.4837 | 0.4615 | 0.5071 |
| DALYs | period_1992 | Both | 1.2778 | 1.2475 | 1.3088 |
| DALYs | period_1997 | Both | 1.1258 | 1.1023 | 1.1498 |
| DALYs | period_2002 | Both | 1 | 1 | 1 |
| DALYs | period_2007 | Both | 0.7803 | 0.764 | 0.797 |
| DALYs | period_2012 | Both | 0.6337 | 0.6184 | 0.6493 |
| DALYs | period_2017 | Both | 0.4765 | 0.4631 | 0.4903 |
| DALYs | period_1992 | Female | 1.3826 | 1.3538 | 1.4121 |
| DALYs | period_1997 | Female | 1.1757 | 1.1545 | 1.1973 |
| DALYs | period_2002 | Female | 1 | 1 | 1 |
| DALYs | period_2007 | Female | 0.7405 | 0.7269 | 0.7544 |
| DALYs | period_2012 | Female | 0.5732 | 0.5609 | 0.5858 |
| DALYs | period_2017 | Female | 0.4345 | 0.4235 | 0.4459 |
| DALYs | period_1992 | Male | 1.2114 | 1.1761 | 1.2478 |
| DALYs | period_1997 | Male | 1.0918 | 1.0637 | 1.1207 |
| DALYs | period_2002 | Male | 1 | 1 | 1 |
| DALYs | period_2007 | Male | 0.8078 | 0.7872 | 0.829 |
| DALYs | period_2012 | Male | 0.6759 | 0.6561 | 0.6962 |
| DALYs | period_2017 | Male | 0.505 | 0.4877 | 0.5229 |
