# Supplementary material for: Trends and projections of PM2.5-attributable disease burden in China: a GBD 2021-based analysis
Source: Front Public Health. 2026 Jan 15;14:1684344. doi: 10.3389/fpubh.2026.1684344 (PMC12852448; doi:10.3389/fpubh.2026.1684344)
Supplement: Supplementary file 17 [file Table_9.DOCX]

| **Table. Relative risk for APMP Mortality rate and DALYs rate of each period compared with the reference (2002–2006)** | | | | | |
| --- | --- | --- | --- | --- | --- |
| **Measure** | **Period** | **Sex** | **Rate Ratio** | **95%CI_Low** | **95%CI_High** |
| Mortality | period_1992 | Both | 0.7601 | 0.7344 | 0.7867 |
| Mortality | period_1997 | Both | 0.8398 | 0.8187 | 0.8614 |
| Mortality | period_2002 | Both | 1 | 1 | 1 |
| Mortality | period_2007 | Both | 1.0503 | 1.0267 | 1.0743 |
| Mortality | period_2012 | Both | 1.1343 | 1.1001 | 1.1695 |
| Mortality | period_2017 | Both | 0.953 | 0.9149 | 0.9927 |
| Mortality | period_1992 | Female | 0.7963 | 0.7662 | 0.8275 |
| Mortality | period_1997 | Female | 0.858 | 0.8349 | 0.8817 |
| Mortality | period_2002 | Female | 1 | 1 | 1 |
| Mortality | period_2007 | Female | 1.0207 | 0.996 | 1.046 |
| Mortality | period_2012 | Female | 1.0632 | 1.0267 | 1.101 |
| Mortality | period_2017 | Female | 0.8987 | 0.8568 | 0.9426 |
| Mortality | period_1992 | Male | 0.7404 | 0.7099 | 0.7722 |
| Mortality | period_1997 | Male | 0.8277 | 0.8018 | 0.8544 |
| Mortality | period_2002 | Male | 1 | 1 | 1 |
| Mortality | period_2007 | Male | 1.0644 | 1.0348 | 1.0948 |
| Mortality | period_2012 | Male | 1.1662 | 1.1237 | 1.2103 |
| Mortality | period_2017 | Male | 0.9696 | 0.9233 | 1.0183 |
| DALYs | period_1992 | Both | 0.7502 | 0.7289 | 0.772 |
| DALYs | period_1997 | Both | 0.8367 | 0.816 | 0.8578 |
| DALYs | period_2002 | Both | 1 | 1 | 1 |
| DALYs | period_2007 | Both | 1.0761 | 1.0528 | 1.0999 |
| DALYs | period_2012 | Both | 1.1898 | 1.1616 | 1.2187 |
| DALYs | period_2017 | Both | 1.0148 | 0.9868 | 1.0435 |
| DALYs | period_1992 | Female | 0.7686 | 0.747 | 0.791 |
| DALYs | period_1997 | Female | 0.8458 | 0.8251 | 0.867 |
| DALYs | period_2002 | Female | 1 | 1 | 1 |
| DALYs | period_2007 | Female | 1.0563 | 1.0336 | 1.0794 |
| DALYs | period_2012 | Female | 1.1516 | 1.1244 | 1.1795 |
| DALYs | period_2017 | Female | 1.0044 | 0.9766 | 1.0329 |
| DALYs | period_1992 | Male | 0.7371 | 0.7128 | 0.7623 |
| DALYs | period_1997 | Male | 0.8294 | 0.8056 | 0.8539 |
| DALYs | period_2002 | Male | 1 | 1 | 1 |
| DALYs | period_2007 | Male | 1.087 | 1.0596 | 1.115 |
| DALYs | period_2012 | Male | 1.2086 | 1.175 | 1.2432 |
| DALYs | period_2017 | Male | 1.0142 | 0.9812 | 1.0483 |
