# Supplementary material for: Trends and projections of PM2.5-attributable disease burden in China: a GBD 2021-based analysis
Source: Front Public Health. 2026 Jan 15;14:1684344. doi: 10.3389/fpubh.2026.1684344 (PMC12852448; doi:10.3389/fpubh.2026.1684344)
Supplement: Supplementary file 18 [file Table_10.DOCX]

| **Table S10. Relative risk for HAP-SF Mortality rate and DALYs rate of each period compared with the reference (2002–2006)** | | | | | |
| --- | --- | --- | --- | --- | --- |
| **Measure** | **Period** | **Sex** | **Rate Ratio** | **95%CI_Low** | **95%CI_High** |
| Mortality | period_1992 | Both | 1.6454 | 1.6003 | 1.6919 |
| Mortality | period_1997 | Both | 1.3196 | 1.2958 | 1.3437 |
| Mortality | period_2002 | Both | 1 | 1 | 1 |
| Mortality | period_2007 | Both | 0.5835 | 0.5724 | 0.5948 |
| Mortality | period_2012 | Both | 0.2806 | 0.2723 | 0.289 |
| Mortality | period_2017 | Both | 0.1376 | 0.132 | 0.1434 |
| Mortality | period_1992 | Female | 1.7575 | 1.7046 | 1.8121 |
| Mortality | period_1997 | Female | 1.3676 | 1.3417 | 1.394 |
| Mortality | period_2002 | Female | 1 | 1 | 1 |
| Mortality | period_2007 | Female | 0.5632 | 0.552 | 0.5747 |
| Mortality | period_2012 | Female | 0.264 | 0.2556 | 0.2727 |
| Mortality | period_2017 | Female | 0.1312 | 0.1253 | 0.1373 |
| Mortality | period_1992 | Male | 1.5672 | 1.5148 | 1.6215 |
| Mortality | period_1997 | Male | 1.2819 | 1.2529 | 1.3117 |
| Mortality | period_2002 | Male | 1 | 1 | 1 |
| Mortality | period_2007 | Male | 0.5983 | 0.584 | 0.613 |
| Mortality | period_2012 | Male | 0.2918 | 0.2813 | 0.3027 |
| Mortality | period_2017 | Male | 0.1405 | 0.1336 | 0.1478 |
| DALYs | period_1992 | Both | 1.6164 | 1.5826 | 1.6509 |
| DALYs | period_1997 | Both | 1.3095 | 1.2871 | 1.3323 |
| DALYs | period_2002 | Both | 1 | 1 | 1 |
| DALYs | period_2007 | Both | 0.5966 | 0.5855 | 0.608 |
| DALYs | period_2012 | Both | 0.2945 | 0.2872 | 0.3019 |
| DALYs | period_2017 | Both | 0.1485 | 0.1438 | 0.1533 |
| DALYs | period_1992 | Female | 1.6896 | 1.657 | 1.7229 |
| DALYs | period_1997 | Female | 1.3419 | 1.3211 | 1.363 |
| DALYs | period_2002 | Female | 1 | 1 | 1 |
| DALYs | period_2007 | Female | 0.5814 | 0.5715 | 0.5914 |
| DALYs | period_2012 | Female | 0.2847 | 0.2783 | 0.2913 |
| DALYs | period_2017 | Female | 0.1478 | 0.1435 | 0.1522 |
| DALYs | period_1992 | Male | 1.5589 | 1.5199 | 1.599 |
| DALYs | period_1997 | Male | 1.2825 | 1.256 | 1.3097 |
| DALYs | period_2002 | Male | 1 | 1 | 1 |
| DALYs | period_2007 | Male | 0.6087 | 0.595 | 0.6228 |
| DALYs | period_2012 | Male | 0.3017 | 0.2928 | 0.3109 |
| DALYs | period_2017 | Male | 0.148 | 0.1424 | 0.1539 |
