# Supplementary material for: Trends and projections of PM2.5-attributable disease burden in China: a GBD 2021-based analysis
Source: Front Public Health. 2026 Jan 15;14:1684344. doi: 10.3389/fpubh.2026.1684344 (PMC12852448; doi:10.3389/fpubh.2026.1684344)
Supplement: Supplementary file 19 [file Table_11.DOCX]

| **Table S11. Relative risk for PMP Mortality rate and DALYs rate of each birth cohort compared with the reference (cohort 1957-1961)** | | | | | |
| --- | --- | --- | --- | --- | --- |
| **Measure** | **Cohort** | **Sex** | **Rate Ratio** | **95%CI_Low** | **95%CI_High** |
| Mortality | cohort_1897 | Both | 9.2004 | 7.5117 | 11.2689 |
| Mortality | cohort_1902 | Both | 7.7663 | 7.1952 | 8.3827 |
| Mortality | cohort_1907 | Both | 6.6412 | 6.3249 | 6.9733 |
| Mortality | cohort_1912 | Both | 5.8377 | 5.6017 | 6.0837 |
| Mortality | cohort_1917 | Both | 5.0511 | 4.8609 | 5.2487 |
| Mortality | cohort_1922 | Both | 4.1646 | 4.0132 | 4.3217 |
| Mortality | cohort_1927 | Both | 3.4569 | 3.3339 | 3.5845 |
| Mortality | cohort_1932 | Both | 2.8444 | 2.7454 | 2.9469 |
| Mortality | cohort_1937 | Both | 2.3555 | 2.2746 | 2.4394 |
| Mortality | cohort_1942 | Both | 1.8826 | 1.8178 | 1.9497 |
| Mortality | cohort_1947 | Both | 1.5274 | 1.4748 | 1.5818 |
| Mortality | cohort_1952 | Both | 1.224 | 1.1814 | 1.2681 |
| Mortality | cohort_1957 | Both | 1 | 1 | 1 |
| Mortality | cohort_1962 | Both | 0.8011 | 0.766 | 0.8379 |
| Mortality | cohort_1967 | Both | 0.7036 | 0.6684 | 0.7408 |
| Mortality | cohort_1972 | Both | 0.5721 | 0.534 | 0.6129 |
| Mortality | cohort_1977 | Both | 0.4931 | 0.4474 | 0.5435 |
| Mortality | cohort_1982 | Both | 0.4432 | 0.3873 | 0.5072 |
| Mortality | cohort_1987 | Both | 0.3838 | 0.3231 | 0.4559 |
| Mortality | cohort_1992 | Both | 0.2602 | 0.2041 | 0.3317 |
| Mortality | cohort_1997 | Both | 0.1794 | 0.1397 | 0.2302 |
| Mortality | cohort_2002 | Both | 0.1047 | 0.0812 | 0.1351 |
| Mortality | cohort_2007 | Both | 0.0629 | 0.0484 | 0.0818 |
| Mortality | cohort_2012 | Both | 0.041 | 0.0312 | 0.0538 |
| Mortality | cohort_2017 | Both | 0.0222 | 0.0166 | 0.0299 |
| Mortality | cohort_1897 | Female | 12.9758 | 11.2402 | 14.9793 |
| Mortality | cohort_1902 | Female | 10.8624 | 10.1682 | 11.604 |
| Mortality | cohort_1907 | Female | 9.4371 | 9.005 | 9.8899 |
| Mortality | cohort_1912 | Female | 7.9171 | 7.5948 | 8.2531 |
| Mortality | cohort_1917 | Female | 6.5857 | 6.3299 | 6.8519 |
| Mortality | cohort_1922 | Female | 5.3557 | 5.1524 | 5.5669 |
| Mortality | cohort_1927 | Female | 4.3601 | 4.1969 | 4.5296 |
| Mortality | cohort_1932 | Female | 3.4934 | 3.3647 | 3.627 |
| Mortality | cohort_1937 | Female | 2.8209 | 2.7179 | 2.9278 |
| Mortality | cohort_1942 | Female | 2.1895 | 2.1092 | 2.2728 |
| Mortality | cohort_1947 | Female | 1.7059 | 1.6431 | 1.7711 |
| Mortality | cohort_1952 | Female | 1.3097 | 1.2608 | 1.3606 |
| Mortality | cohort_1957 | Female | 1 | 1 | 1 |
| Mortality | cohort_1962 | Female | 0.7507 | 0.7146 | 0.7887 |
| Mortality | cohort_1967 | Female | 0.6064 | 0.5726 | 0.6422 |
| Mortality | cohort_1972 | Female | 0.4648 | 0.4298 | 0.5027 |
| Mortality | cohort_1977 | Female | 0.3619 | 0.3232 | 0.4052 |
| Mortality | cohort_1982 | Female | 0.2916 | 0.2488 | 0.3418 |
| Mortality | cohort_1987 | Female | 0.2274 | 0.1861 | 0.278 |
| Mortality | cohort_1992 | Female | 0.1489 | 0.1138 | 0.1948 |
| Mortality | cohort_1997 | Female | 0.1012 | 0.0769 | 0.1331 |
| Mortality | cohort_2002 | Female | 0.0578 | 0.0437 | 0.0764 |
| Mortality | cohort_2007 | Female | 0.0341 | 0.0256 | 0.0454 |
| Mortality | cohort_2012 | Female | 0.022 | 0.0164 | 0.0296 |
| Mortality | cohort_2017 | Female | 0.0114 | 0.0083 | 0.0157 |
| Mortality | cohort_1897 | Male | 5.7093 | 3.4089 | 9.5621 |
| Mortality | cohort_1902 | Male | 5.8827 | 5.2015 | 6.6531 |
| Mortality | cohort_1907 | Male | 5.1282 | 4.7804 | 5.5013 |
| Mortality | cohort_1912 | Male | 4.7786 | 4.5161 | 5.0564 |
| Mortality | cohort_1917 | Male | 4.313 | 4.0983 | 4.539 |
| Mortality | cohort_1922 | Male | 3.5592 | 3.3903 | 3.7365 |
| Mortality | cohort_1927 | Male | 2.9807 | 2.8434 | 3.1247 |
| Mortality | cohort_1932 | Male | 2.4925 | 2.3807 | 2.6095 |
| Mortality | cohort_1937 | Male | 2.0969 | 2.0043 | 2.1939 |
| Mortality | cohort_1942 | Male | 1.7092 | 1.6338 | 1.7882 |
| Mortality | cohort_1947 | Male | 1.4281 | 1.3652 | 1.4938 |
| Mortality | cohort_1952 | Male | 1.1807 | 1.1284 | 1.2355 |
| Mortality | cohort_1957 | Male | 1 | 1 | 1 |
| Mortality | cohort_1962 | Male | 0.8325 | 0.7866 | 0.8811 |
| Mortality | cohort_1967 | Male | 0.7635 | 0.7156 | 0.8145 |
| Mortality | cohort_1972 | Male | 0.6366 | 0.5841 | 0.6938 |
| Mortality | cohort_1977 | Male | 0.5764 | 0.5111 | 0.6501 |
| Mortality | cohort_1982 | Male | 0.545 | 0.4616 | 0.6434 |
| Mortality | cohort_1987 | Male | 0.4984 | 0.4028 | 0.6169 |
| Mortality | cohort_1992 | Male | 0.3446 | 0.2532 | 0.4689 |
| Mortality | cohort_1997 | Male | 0.2401 | 0.1747 | 0.3302 |
| Mortality | cohort_2002 | Male | 0.1418 | 0.1025 | 0.1963 |
| Mortality | cohort_2007 | Male | 0.0863 | 0.0617 | 0.1207 |
| Mortality | cohort_2012 | Male | 0.0568 | 0.04 | 0.0804 |
| Mortality | cohort_2017 | Male | 0.0317 | 0.0217 | 0.0464 |
| DALYs | cohort_1897 | Both | 8.3862 | 5.6441 | 12.4604 |
| DALYs | cohort_1902 | Both | 7.0813 | 6.1818 | 8.1115 |
| DALYs | cohort_1907 | Both | 6.0657 | 5.6454 | 6.5174 |
| DALYs | cohort_1912 | Both | 5.3443 | 5.0761 | 5.6267 |
| DALYs | cohort_1917 | Both | 4.634 | 4.4387 | 4.838 |
| DALYs | cohort_1922 | Both | 3.8652 | 3.717 | 4.0192 |
| DALYs | cohort_1927 | Both | 3.2329 | 3.1157 | 3.3544 |
| DALYs | cohort_1932 | Both | 2.6785 | 2.5859 | 2.7743 |
| DALYs | cohort_1937 | Both | 2.2264 | 2.1517 | 2.3036 |
| DALYs | cohort_1942 | Both | 1.8096 | 1.7496 | 1.8715 |
| DALYs | cohort_1947 | Both | 1.4898 | 1.4414 | 1.5399 |
| DALYs | cohort_1952 | Both | 1.2108 | 1.1719 | 1.251 |
| DALYs | cohort_1957 | Both | 1 | 1 | 1 |
| DALYs | cohort_1962 | Both | 0.8244 | 0.7932 | 0.8568 |
| DALYs | cohort_1967 | Both | 0.7284 | 0.6982 | 0.76 |
| DALYs | cohort_1972 | Both | 0.6092 | 0.5775 | 0.6428 |
| DALYs | cohort_1977 | Both | 0.5376 | 0.5006 | 0.5774 |
| DALYs | cohort_1982 | Both | 0.4974 | 0.4532 | 0.5458 |
| DALYs | cohort_1987 | Both | 0.4478 | 0.4001 | 0.5012 |
| DALYs | cohort_1992 | Both | 0.3165 | 0.2715 | 0.369 |
| DALYs | cohort_1997 | Both | 0.2189 | 0.1868 | 0.2564 |
| DALYs | cohort_2002 | Both | 0.129 | 0.1098 | 0.1515 |
| DALYs | cohort_2007 | Both | 0.0772 | 0.0654 | 0.091 |
| DALYs | cohort_2012 | Both | 0.0501 | 0.0423 | 0.0595 |
| DALYs | cohort_2017 | Both | 0.0272 | 0.0226 | 0.0327 |
| DALYs | cohort_1897 | Female | 11.2088 | 8.7765 | 14.3151 |
| DALYs | cohort_1902 | Female | 9.3552 | 8.4757 | 10.3261 |
| DALYs | cohort_1907 | Female | 8.1482 | 7.7004 | 8.6221 |
| DALYs | cohort_1912 | Female | 6.8554 | 6.5682 | 7.1552 |
| DALYs | cohort_1917 | Female | 5.7597 | 5.5487 | 5.9788 |
| DALYs | cohort_1922 | Female | 4.7424 | 4.5807 | 4.9098 |
| DALYs | cohort_1927 | Female | 3.8943 | 3.7668 | 4.0261 |
| DALYs | cohort_1932 | Female | 3.1584 | 3.0588 | 3.2612 |
| DALYs | cohort_1937 | Female | 2.5759 | 2.4966 | 2.6577 |
| DALYs | cohort_1942 | Female | 2.0457 | 1.9833 | 2.1101 |
| DALYs | cohort_1947 | Female | 1.6299 | 1.5808 | 1.6804 |
| DALYs | cohort_1952 | Female | 1.2801 | 1.2419 | 1.3195 |
| DALYs | cohort_1957 | Female | 1 | 1 | 1 |
| DALYs | cohort_1962 | Female | 0.7842 | 0.7563 | 0.8133 |
| DALYs | cohort_1967 | Female | 0.6492 | 0.6235 | 0.6759 |
| DALYs | cohort_1972 | Female | 0.5233 | 0.4972 | 0.5508 |
| DALYs | cohort_1977 | Female | 0.4316 | 0.4029 | 0.4623 |
| DALYs | cohort_1982 | Female | 0.374 | 0.342 | 0.409 |
| DALYs | cohort_1987 | Female | 0.3171 | 0.2851 | 0.3529 |
| DALYs | cohort_1992 | Female | 0.2205 | 0.1913 | 0.2541 |
| DALYs | cohort_1997 | Female | 0.1502 | 0.1298 | 0.1738 |
| DALYs | cohort_2002 | Female | 0.0867 | 0.0748 | 0.1006 |
| DALYs | cohort_2007 | Female | 0.0512 | 0.0439 | 0.0596 |
| DALYs | cohort_2012 | Female | 0.0328 | 0.028 | 0.0384 |
| DALYs | cohort_2017 | Female | 0.017 | 0.0144 | 0.0202 |
| DALYs | cohort_1897 | Male | 5.3736 | 2.1385 | 13.503 |
| DALYs | cohort_1902 | Male | 5.5504 | 4.523 | 6.8111 |
| DALYs | cohort_1907 | Male | 4.8299 | 4.3755 | 5.3315 |
| DALYs | cohort_1912 | Male | 4.5044 | 4.2117 | 4.8174 |
| DALYs | cohort_1917 | Male | 4.0432 | 3.8315 | 4.2666 |
| DALYs | cohort_1922 | Male | 3.3832 | 3.2259 | 3.5483 |
| DALYs | cohort_1927 | Male | 2.8557 | 2.7319 | 2.9852 |
| DALYs | cohort_1932 | Male | 2.395 | 2.2968 | 2.4974 |
| DALYs | cohort_1937 | Male | 2.0134 | 1.9336 | 2.0965 |
| DALYs | cohort_1942 | Male | 1.6639 | 1.599 | 1.7315 |
| DALYs | cohort_1947 | Male | 1.4051 | 1.3515 | 1.4609 |
| DALYs | cohort_1952 | Male | 1.1731 | 1.1289 | 1.2189 |
| DALYs | cohort_1957 | Male | 1 | 1 | 1 |
| DALYs | cohort_1962 | Male | 0.8514 | 0.814 | 0.8905 |
| DALYs | cohort_1967 | Male | 0.7811 | 0.7435 | 0.8205 |
| DALYs | cohort_1972 | Male | 0.6655 | 0.6256 | 0.7081 |
| DALYs | cohort_1977 | Male | 0.6113 | 0.5631 | 0.6637 |
| DALYs | cohort_1982 | Male | 0.5883 | 0.5286 | 0.6549 |
| DALYs | cohort_1987 | Male | 0.5514 | 0.4837 | 0.6285 |
| DALYs | cohort_1992 | Male | 0.3923 | 0.3274 | 0.4701 |
| DALYs | cohort_1997 | Male | 0.274 | 0.2273 | 0.3304 |
| DALYs | cohort_2002 | Male | 0.1636 | 0.1352 | 0.1978 |
| DALYs | cohort_2007 | Male | 0.0988 | 0.0813 | 0.1201 |
| DALYs | cohort_2012 | Male | 0.0649 | 0.0531 | 0.0795 |
| DALYs | cohort_2017 | Male | 0.0363 | 0.0292 | 0.0452 |
