# Supplementary material for: Trends and projections of PM2.5-attributable disease burden in China: a GBD 2021-based analysis
Source: Front Public Health. 2026 Jan 15;14:1684344. doi: 10.3389/fpubh.2026.1684344 (PMC12852448; doi:10.3389/fpubh.2026.1684344)
Supplement: Supplementary file 20 [file Table_12.DOCX]

| **Table S12. Relative risk for APMP Mortality rate and DALYs rate of each birth cohort compared with the reference (cohort 1957-1961)** | | | | | |
| --- | --- | --- | --- | --- | --- |
| **Measure** | **Cohort** | **Sex** | **Rate Ratio** | **95%CI_Low** | **95%CI_High** |
| Mortality | cohort_1897 | Both | 0.3769 | 0.2732 | 0.5201 |
| Mortality | cohort_1902 | Both | 0.424 | 0.3797 | 0.4734 |
| Mortality | cohort_1907 | Both | 0.4695 | 0.4407 | 0.5002 |
| Mortality | cohort_1912 | Both | 0.5336 | 0.5075 | 0.5611 |
| Mortality | cohort_1917 | Both | 0.6006 | 0.574 | 0.6284 |
| Mortality | cohort_1922 | Both | 0.6451 | 0.6181 | 0.6733 |
| Mortality | cohort_1927 | Both | 0.6901 | 0.6624 | 0.7189 |
| Mortality | cohort_1932 | Both | 0.7344 | 0.706 | 0.764 |
| Mortality | cohort_1937 | Both | 0.7868 | 0.7571 | 0.8176 |
| Mortality | cohort_1942 | Both | 0.8168 | 0.7865 | 0.8483 |
| Mortality | cohort_1947 | Both | 0.8826 | 0.8505 | 0.9158 |
| Mortality | cohort_1952 | Both | 0.9336 | 0.9003 | 0.9681 |
| Mortality | cohort_1957 | Both | 1 | 1 | 1 |
| Mortality | cohort_1962 | Both | 1.0429 | 0.9964 | 1.0916 |
| Mortality | cohort_1967 | Both | 1.1709 | 1.1095 | 1.2357 |
| Mortality | cohort_1972 | Both | 1.2224 | 1.1373 | 1.3138 |
| Mortality | cohort_1977 | Both | 1.3991 | 1.2661 | 1.5461 |
| Mortality | cohort_1982 | Both | 1.7001 | 1.482 | 1.9505 |
| Mortality | cohort_1987 | Both | 1.9882 | 1.6614 | 2.3792 |
| Mortality | cohort_1992 | Both | 1.9567 | 1.4911 | 2.5678 |
| Mortality | cohort_1997 | Both | 1.679 | 1.2548 | 2.2466 |
| Mortality | cohort_2002 | Both | 1.2639 | 0.9397 | 1.7 |
| Mortality | cohort_2007 | Both | 1.0505 | 0.7778 | 1.4188 |
| Mortality | cohort_2012 | Both | 0.9964 | 0.7367 | 1.3476 |
| Mortality | cohort_2017 | Both | 0.7344 | 0.535 | 1.0081 |
| Mortality | cohort_1897 | Female | 0.3983 | 0.3144 | 0.5046 |
| Mortality | cohort_1902 | Female | 0.4335 | 0.3927 | 0.4785 |
| Mortality | cohort_1907 | Female | 0.4944 | 0.4642 | 0.5265 |
| Mortality | cohort_1912 | Female | 0.5491 | 0.5208 | 0.579 |
| Mortality | cohort_1917 | Female | 0.6005 | 0.5715 | 0.6309 |
| Mortality | cohort_1922 | Female | 0.6527 | 0.6224 | 0.6843 |
| Mortality | cohort_1927 | Female | 0.7044 | 0.6728 | 0.7375 |
| Mortality | cohort_1932 | Female | 0.7494 | 0.7168 | 0.7836 |
| Mortality | cohort_1937 | Female | 0.8026 | 0.7684 | 0.8383 |
| Mortality | cohort_1942 | Female | 0.841 | 0.8055 | 0.878 |
| Mortality | cohort_1947 | Female | 0.905 | 0.8675 | 0.9442 |
| Mortality | cohort_1952 | Female | 0.9575 | 0.9183 | 0.9983 |
| Mortality | cohort_1957 | Female | 1 | 1 | 1 |
| Mortality | cohort_1962 | Female | 1.0173 | 0.964 | 1.0734 |
| Mortality | cohort_1967 | Female | 1.0892 | 1.0212 | 1.1619 |
| Mortality | cohort_1972 | Female | 1.1192 | 1.0249 | 1.2223 |
| Mortality | cohort_1977 | Female | 1.1978 | 1.0573 | 1.3569 |
| Mortality | cohort_1982 | Female | 1.3443 | 1.1294 | 1.6002 |
| Mortality | cohort_1987 | Female | 1.463 | 1.1683 | 1.832 |
| Mortality | cohort_1992 | Female | 1.3309 | 0.9638 | 1.8379 |
| Mortality | cohort_1997 | Female | 1.1493 | 0.8189 | 1.6129 |
| Mortality | cohort_2002 | Female | 0.8638 | 0.6123 | 1.2186 |
| Mortality | cohort_2007 | Female | 0.7181 | 0.507 | 1.017 |
| Mortality | cohort_2012 | Female | 0.6868 | 0.4842 | 0.9742 |
| Mortality | cohort_2017 | Female | 0.4958 | 0.3447 | 0.7131 |
| Mortality | cohort_1897 | Male | 0.3218 | 0.1603 | 0.6456 |
| Mortality | cohort_1902 | Male | 0.4231 | 0.3597 | 0.4977 |
| Mortality | cohort_1907 | Male | 0.4634 | 0.4256 | 0.5045 |
| Mortality | cohort_1912 | Male | 0.5407 | 0.5071 | 0.5765 |
| Mortality | cohort_1917 | Male | 0.62 | 0.5861 | 0.6557 |
| Mortality | cohort_1922 | Male | 0.6447 | 0.6119 | 0.6793 |
| Mortality | cohort_1927 | Male | 0.6774 | 0.6446 | 0.712 |
| Mortality | cohort_1932 | Male | 0.7165 | 0.6832 | 0.7514 |
| Mortality | cohort_1937 | Male | 0.7647 | 0.7301 | 0.8009 |
| Mortality | cohort_1942 | Male | 0.792 | 0.7569 | 0.8289 |
| Mortality | cohort_1947 | Male | 0.864 | 0.8266 | 0.9031 |
| Mortality | cohort_1952 | Male | 0.9223 | 0.8832 | 0.9631 |
| Mortality | cohort_1957 | Male | 1 | 1 | 1 |
| Mortality | cohort_1962 | Male | 1.0612 | 1.0056 | 1.1199 |
| Mortality | cohort_1967 | Male | 1.2198 | 1.1451 | 1.2994 |
| Mortality | cohort_1972 | Male | 1.2795 | 1.1765 | 1.3916 |
| Mortality | cohort_1977 | Male | 1.5069 | 1.3428 | 1.6911 |
| Mortality | cohort_1982 | Male | 1.8875 | 1.6121 | 2.2101 |
| Mortality | cohort_1987 | Male | 2.2779 | 1.8516 | 2.8023 |
| Mortality | cohort_1992 | Male | 2.3151 | 1.6803 | 3.1896 |
| Mortality | cohort_1997 | Male | 1.9749 | 1.3951 | 2.7957 |
| Mortality | cohort_2002 | Male | 1.4791 | 1.0379 | 2.108 |
| Mortality | cohort_2007 | Male | 1.2305 | 0.8588 | 1.7632 |
| Mortality | cohort_2012 | Male | 1.1669 | 0.813 | 1.6749 |
| Mortality | cohort_2017 | Male | 0.8737 | 0.5968 | 1.2789 |
| DALYs | cohort_1897 | Both | 0.3469 | 0.1982 | 0.607 |
| DALYs | cohort_1902 | Both | 0.3909 | 0.3265 | 0.4681 |
| DALYs | cohort_1907 | Both | 0.4337 | 0.3968 | 0.474 |
| DALYs | cohort_1912 | Both | 0.4937 | 0.4652 | 0.5239 |
| DALYs | cohort_1917 | Both | 0.5565 | 0.5308 | 0.5835 |
| DALYs | cohort_1922 | Both | 0.6048 | 0.5802 | 0.6305 |
| DALYs | cohort_1927 | Both | 0.6534 | 0.6288 | 0.679 |
| DALYs | cohort_1932 | Both | 0.6991 | 0.6744 | 0.7247 |
| DALYs | cohort_1937 | Both | 0.748 | 0.7228 | 0.7741 |
| DALYs | cohort_1942 | Both | 0.7872 | 0.7615 | 0.8138 |
| DALYs | cohort_1947 | Both | 0.8638 | 0.8369 | 0.8917 |
| DALYs | cohort_1952 | Both | 0.925 | 0.8973 | 0.9536 |
| DALYs | cohort_1957 | Both | 1 | 1 | 1 |
| DALYs | cohort_1962 | Both | 1.0719 | 1.0345 | 1.1107 |
| DALYs | cohort_1967 | Both | 1.2113 | 1.1636 | 1.2609 |
| DALYs | cohort_1972 | Both | 1.3009 | 1.2365 | 1.3686 |
| DALYs | cohort_1977 | Both | 1.5214 | 1.4238 | 1.6257 |
| DALYs | cohort_1982 | Both | 1.9 | 1.7447 | 2.069 |
| DALYs | cohort_1987 | Both | 2.2989 | 2.0696 | 2.5536 |
| DALYs | cohort_1992 | Both | 2.3857 | 2.0515 | 2.7744 |
| DALYs | cohort_1997 | Both | 2.0602 | 1.752 | 2.4226 |
| DALYs | cohort_2002 | Both | 1.5713 | 1.3336 | 1.8513 |
| DALYs | cohort_2007 | Both | 1.2971 | 1.0991 | 1.5309 |
| DALYs | cohort_2012 | Both | 1.2231 | 1.0357 | 1.4446 |
| DALYs | cohort_2017 | Both | 0.9026 | 0.7579 | 1.0749 |
| DALYs | cohort_1897 | Female | 0.3521 | 0.241 | 0.5145 |
| DALYs | cohort_1902 | Female | 0.3825 | 0.3308 | 0.4424 |
| DALYs | cohort_1907 | Female | 0.4376 | 0.4051 | 0.4728 |
| DALYs | cohort_1912 | Female | 0.4867 | 0.4607 | 0.514 |
| DALYs | cohort_1917 | Female | 0.5378 | 0.5137 | 0.5631 |
| DALYs | cohort_1922 | Female | 0.5917 | 0.5676 | 0.6168 |
| DALYs | cohort_1927 | Female | 0.6449 | 0.6202 | 0.6705 |
| DALYs | cohort_1932 | Female | 0.6924 | 0.6673 | 0.7184 |
| DALYs | cohort_1937 | Female | 0.7446 | 0.7187 | 0.7714 |
| DALYs | cohort_1942 | Female | 0.7938 | 0.7669 | 0.8217 |
| DALYs | cohort_1947 | Female | 0.8726 | 0.8442 | 0.9019 |
| DALYs | cohort_1952 | Female | 0.94 | 0.9105 | 0.9703 |
| DALYs | cohort_1957 | Female | 1 | 1 | 1 |
| DALYs | cohort_1962 | Female | 1.06 | 1.0207 | 1.1008 |
| DALYs | cohort_1967 | Female | 1.1606 | 1.1115 | 1.2118 |
| DALYs | cohort_1972 | Female | 1.25 | 1.1832 | 1.3206 |
| DALYs | cohort_1977 | Female | 1.4114 | 1.313 | 1.5171 |
| DALYs | cohort_1982 | Female | 1.7001 | 1.5501 | 1.8646 |
| DALYs | cohort_1987 | Female | 1.9971 | 1.7864 | 2.2327 |
| DALYs | cohort_1992 | Female | 1.9897 | 1.7039 | 2.3234 |
| DALYs | cohort_1997 | Female | 1.7301 | 1.4669 | 2.0405 |
| DALYs | cohort_2002 | Female | 1.3229 | 1.1195 | 1.5631 |
| DALYs | cohort_2007 | Female | 1.0968 | 0.9267 | 1.2981 |
| DALYs | cohort_2012 | Female | 1.035 | 0.874 | 1.2258 |
| DALYs | cohort_2017 | Female | 0.7465 | 0.6256 | 0.8908 |
| DALYs | cohort_1897 | Male | 0.3041 | 0.0974 | 0.9492 |
| DALYs | cohort_1902 | Male | 0.4026 | 0.3129 | 0.5179 |
| DALYs | cohort_1907 | Male | 0.4402 | 0.3922 | 0.494 |
| DALYs | cohort_1912 | Male | 0.5138 | 0.4772 | 0.5531 |
| DALYs | cohort_1917 | Male | 0.5854 | 0.5533 | 0.6193 |
| DALYs | cohort_1922 | Male | 0.6178 | 0.5886 | 0.6484 |
| DALYs | cohort_1927 | Male | 0.6553 | 0.6271 | 0.6849 |
| DALYs | cohort_1932 | Male | 0.6941 | 0.6663 | 0.7231 |
| DALYs | cohort_1937 | Male | 0.7373 | 0.7092 | 0.7666 |
| DALYs | cohort_1942 | Male | 0.7723 | 0.7438 | 0.8019 |
| DALYs | cohort_1947 | Male | 0.8524 | 0.8224 | 0.8835 |
| DALYs | cohort_1952 | Male | 0.9176 | 0.8867 | 0.9496 |
| DALYs | cohort_1957 | Male | 1 | 1 | 1 |
| DALYs | cohort_1962 | Male | 1.084 | 1.0418 | 1.1279 |
| DALYs | cohort_1967 | Male | 1.2475 | 1.193 | 1.3045 |
| DALYs | cohort_1972 | Male | 1.3369 | 1.2638 | 1.4143 |
| DALYs | cohort_1977 | Male | 1.5938 | 1.4811 | 1.7149 |
| DALYs | cohort_1982 | Male | 2.0275 | 1.8448 | 2.2283 |
| DALYs | cohort_1987 | Male | 2.4982 | 2.2211 | 2.8098 |
| DALYs | cohort_1992 | Male | 2.6354 | 2.2195 | 3.1291 |
| DALYs | cohort_1997 | Male | 2.2585 | 1.8764 | 2.7184 |
| DALYs | cohort_2002 | Male | 1.7138 | 1.4206 | 2.0674 |
| DALYs | cohort_2007 | Male | 1.413 | 1.1688 | 1.7081 |
| DALYs | cohort_2012 | Male | 1.3363 | 1.1045 | 1.6168 |
| DALYs | cohort_2017 | Male | 1.002 | 0.8199 | 1.2247 |
