# Supplementary material for: Trends and projections of PM2.5-attributable disease burden in China: a GBD 2021-based analysis
Source: Front Public Health. 2026 Jan 15;14:1684344. doi: 10.3389/fpubh.2026.1684344 (PMC12852448; doi:10.3389/fpubh.2026.1684344)
Supplement: Supplementary file 21 [file Table_13.DOCX]

| **Table S13. Relative risk for HAP-SF Mortality rate and DALYs rate of each birth cohort compared with the reference (cohort 1957-1961)** | | | | | |
| --- | --- | --- | --- | --- | --- |
| **Measure** | **Cohort** | **Sex** | **Rate Ratio** | **95%CI_Low** | **95%CI_High** |
| Mortality | cohort_1897 | Both | 295.5605 | 251.1263 | 347.8569 |
| Mortality | cohort_1902 | Both | 187.4595 | 174.9435 | 200.8711 |
| Mortality | cohort_1907 | Both | 119.8588 | 114.0423 | 125.972 |
| Mortality | cohort_1912 | Both | 78.5006 | 75.1134 | 82.0406 |
| Mortality | cohort_1917 | Both | 50.374 | 48.3257 | 52.5091 |
| Mortality | cohort_1922 | Both | 30.7892 | 29.5795 | 32.0483 |
| Mortality | cohort_1927 | Both | 19.1793 | 18.4421 | 19.9459 |
| Mortality | cohort_1932 | Both | 11.9042 | 11.4551 | 12.371 |
| Mortality | cohort_1937 | Both | 7.5084 | 7.2288 | 7.7988 |
| Mortality | cohort_1942 | Both | 4.5903 | 4.4202 | 4.7669 |
| Mortality | cohort_1947 | Both | 2.7555 | 2.6537 | 2.8611 |
| Mortality | cohort_1952 | Both | 1.6393 | 1.5783 | 1.7025 |
| Mortality | cohort_1957 | Both | 1 | 1 | 1 |
| Mortality | cohort_1962 | Both | 0.6041 | 0.5755 | 0.634 |
| Mortality | cohort_1967 | Both | 0.4068 | 0.3848 | 0.4301 |
| Mortality | cohort_1972 | Both | 0.2504 | 0.2321 | 0.2701 |
| Mortality | cohort_1977 | Both | 0.16 | 0.1432 | 0.1788 |
| Mortality | cohort_1982 | Both | 0.1024 | 0.0873 | 0.1199 |
| Mortality | cohort_1987 | Both | 0.0628 | 0.0512 | 0.0769 |
| Mortality | cohort_1992 | Both | 0.0312 | 0.0241 | 0.0404 |
| Mortality | cohort_1997 | Both | 0.0167 | 0.0129 | 0.0217 |
| Mortality | cohort_2002 | Both | 0.0077 | 0.0059 | 0.0101 |
| Mortality | cohort_2007 | Both | 0.0037 | 0.0028 | 0.0049 |
| Mortality | cohort_2012 | Both | 0.0019 | 0.0015 | 0.0026 |
| Mortality | cohort_2017 | Both | 0.0008 | 0.0006 | 0.0012 |
| Mortality | cohort_1897 | Female | 330.4489 | 291.6718 | 374.3815 |
| Mortality | cohort_1902 | Female | 211.3325 | 198.1481 | 225.3942 |
| Mortality | cohort_1907 | Female | 138.0305 | 131.2816 | 145.1264 |
| Mortality | cohort_1912 | Female | 87.5203 | 83.607 | 91.6167 |
| Mortality | cohort_1917 | Female | 55.0223 | 52.6669 | 57.483 |
| Mortality | cohort_1922 | Female | 33.745 | 32.3361 | 35.2153 |
| Mortality | cohort_1927 | Female | 20.9536 | 20.0921 | 21.8519 |
| Mortality | cohort_1932 | Female | 12.942 | 12.417 | 13.4892 |
| Mortality | cohort_1937 | Female | 8.1725 | 7.844 | 8.5147 |
| Mortality | cohort_1942 | Female | 4.9476 | 4.7492 | 5.1542 |
| Mortality | cohort_1947 | Female | 2.9275 | 2.8102 | 3.0496 |
| Mortality | cohort_1952 | Female | 1.7097 | 1.6406 | 1.7817 |
| Mortality | cohort_1957 | Female | 1 | 1 | 1 |
| Mortality | cohort_1962 | Female | 0.5824 | 0.552 | 0.6145 |
| Mortality | cohort_1967 | Female | 0.3719 | 0.3494 | 0.3958 |
| Mortality | cohort_1972 | Female | 0.2205 | 0.2023 | 0.2404 |
| Mortality | cohort_1977 | Female | 0.1331 | 0.1172 | 0.1511 |
| Mortality | cohort_1982 | Female | 0.081 | 0.0675 | 0.0972 |
| Mortality | cohort_1987 | Female | 0.0469 | 0.0372 | 0.0592 |
| Mortality | cohort_1992 | Female | 0.0236 | 0.0177 | 0.0315 |
| Mortality | cohort_1997 | Female | 0.0126 | 0.0094 | 0.0168 |
| Mortality | cohort_2002 | Female | 0.0057 | 0.0043 | 0.0077 |
| Mortality | cohort_2007 | Female | 0.0027 | 0.002 | 0.0037 |
| Mortality | cohort_2012 | Female | 0.0014 | 0.001 | 0.0019 |
| Mortality | cohort_2017 | Female | 0.0006 | 0.0004 | 0.0008 |
| Mortality | cohort_1897 | Male | 258.3885 | 174.216 | 383.2288 |
| Mortality | cohort_1902 | Male | 165.8254 | 149.7836 | 183.5852 |
| Mortality | cohort_1907 | Male | 106.2777 | 99.4804 | 113.5393 |
| Mortality | cohort_1912 | Male | 73.0438 | 69.024 | 77.2978 |
| Mortality | cohort_1917 | Male | 48.0289 | 45.5847 | 50.6042 |
| Mortality | cohort_1922 | Male | 29.0247 | 27.6123 | 30.5093 |
| Mortality | cohort_1927 | Male | 18.0402 | 17.1871 | 18.9356 |
| Mortality | cohort_1932 | Male | 11.2001 | 10.6827 | 11.7425 |
| Mortality | cohort_1937 | Male | 7.0413 | 6.7215 | 7.3764 |
| Mortality | cohort_1942 | Male | 4.3392 | 4.1435 | 4.5442 |
| Mortality | cohort_1947 | Male | 2.6391 | 2.5207 | 2.763 |
| Mortality | cohort_1952 | Male | 1.5967 | 1.5247 | 1.6721 |
| Mortality | cohort_1957 | Male | 1 | 1 | 1 |
| Mortality | cohort_1962 | Male | 0.6195 | 0.5844 | 0.6567 |
| Mortality | cohort_1967 | Male | 0.4311 | 0.4033 | 0.4607 |
| Mortality | cohort_1972 | Male | 0.2705 | 0.2472 | 0.296 |
| Mortality | cohort_1977 | Male | 0.1792 | 0.1572 | 0.2042 |
| Mortality | cohort_1982 | Male | 0.1182 | 0.098 | 0.1425 |
| Mortality | cohort_1987 | Male | 0.0753 | 0.0592 | 0.0958 |
| Mortality | cohort_1992 | Male | 0.037 | 0.0271 | 0.0505 |
| Mortality | cohort_1997 | Male | 0.0199 | 0.0145 | 0.0273 |
| Mortality | cohort_2002 | Male | 0.0093 | 0.0067 | 0.0128 |
| Mortality | cohort_2007 | Male | 0.0045 | 0.0033 | 0.0063 |
| Mortality | cohort_2012 | Male | 0.0024 | 0.0017 | 0.0034 |
| Mortality | cohort_2017 | Male | 0.001 | 0.0007 | 0.0016 |
| DALYs | cohort_1897 | Both | 250.3664 | 185.4946 | 337.9253 |
| DALYs | cohort_1902 | Both | 160.0427 | 143.4106 | 178.6038 |
| DALYs | cohort_1907 | Both | 103.1588 | 96.761 | 109.9796 |
| DALYs | cohort_1912 | Both | 68.193 | 64.8991 | 71.6539 |
| DALYs | cohort_1917 | Both | 44.1469 | 42.2906 | 46.0848 |
| DALYs | cohort_1922 | Both | 27.5067 | 26.4444 | 28.6118 |
| DALYs | cohort_1927 | Both | 17.3702 | 16.7369 | 18.0274 |
| DALYs | cohort_1932 | Both | 10.941 | 10.5603 | 11.3354 |
| DALYs | cohort_1937 | Both | 6.9759 | 6.7414 | 7.2184 |
| DALYs | cohort_1942 | Both | 4.3488 | 4.206 | 4.4964 |
| DALYs | cohort_1947 | Both | 2.662 | 2.5767 | 2.7501 |
| DALYs | cohort_1952 | Both | 1.6152 | 1.5644 | 1.6677 |
| DALYs | cohort_1957 | Both | 1 | 1 | 1 |
| DALYs | cohort_1962 | Both | 0.6225 | 0.5991 | 0.6467 |
| DALYs | cohort_1967 | Both | 0.422 | 0.4045 | 0.4403 |
| DALYs | cohort_1972 | Both | 0.2687 | 0.2544 | 0.2838 |
| DALYs | cohort_1977 | Both | 0.1763 | 0.1635 | 0.1901 |
| DALYs | cohort_1982 | Both | 0.116 | 0.1047 | 0.1285 |
| DALYs | cohort_1987 | Both | 0.0734 | 0.0647 | 0.0833 |
| DALYs | cohort_1992 | Both | 0.0372 | 0.0318 | 0.0436 |
| DALYs | cohort_1997 | Both | 0.0201 | 0.0171 | 0.0236 |
| DALYs | cohort_2002 | Both | 0.0094 | 0.008 | 0.0111 |
| DALYs | cohort_2007 | Both | 0.0045 | 0.0038 | 0.0054 |
| DALYs | cohort_2012 | Both | 0.0024 | 0.002 | 0.0028 |
| DALYs | cohort_2017 | Both | 0.001 | 0.0008 | 0.0012 |
| DALYs | cohort_1897 | Female | 262.0095 | 214.7517 | 319.6668 |
| DALYs | cohort_1902 | Female | 168.5353 | 154.7152 | 183.5899 |
| DALYs | cohort_1907 | Female | 111.1909 | 105.3765 | 117.3261 |
| DALYs | cohort_1912 | Female | 71.3414 | 68.328 | 74.4877 |
| DALYs | cohort_1917 | Female | 45.6312 | 43.9098 | 47.42 |
| DALYs | cohort_1922 | Female | 28.6105 | 27.6031 | 29.6546 |
| DALYs | cohort_1927 | Female | 18.0725 | 17.4648 | 18.7013 |
| DALYs | cohort_1932 | Female | 11.3979 | 11.0288 | 11.7793 |
| DALYs | cohort_1937 | Female | 7.3214 | 7.091 | 7.5593 |
| DALYs | cohort_1942 | Female | 4.5536 | 4.413 | 4.6987 |
| DALYs | cohort_1947 | Female | 2.7712 | 2.6874 | 2.8576 |
| DALYs | cohort_1952 | Female | 1.6651 | 1.6155 | 1.7162 |
| DALYs | cohort_1957 | Female | 1 | 1 | 1 |
| DALYs | cohort_1962 | Female | 0.6082 | 0.5863 | 0.6308 |
| DALYs | cohort_1967 | Female | 0.3979 | 0.382 | 0.4145 |
| DALYs | cohort_1972 | Female | 0.2494 | 0.2366 | 0.2629 |
| DALYs | cohort_1977 | Female | 0.1587 | 0.1475 | 0.1706 |
| DALYs | cohort_1982 | Female | 0.1024 | 0.0928 | 0.1129 |
| DALYs | cohort_1987 | Female | 0.0632 | 0.0561 | 0.0713 |
| DALYs | cohort_1992 | Female | 0.0327 | 0.0281 | 0.038 |
| DALYs | cohort_1997 | Female | 0.0176 | 0.0151 | 0.0205 |
| DALYs | cohort_2002 | Female | 0.0081 | 0.007 | 0.0095 |
| DALYs | cohort_2007 | Female | 0.0039 | 0.0033 | 0.0045 |
| DALYs | cohort_2012 | Female | 0.002 | 0.0017 | 0.0023 |
| DALYs | cohort_2017 | Female | 0.0008 | 0.0007 | 0.001 |
| DALYs | cohort_1897 | Male | 231.2112 | 114.7367 | 465.9243 |
| DALYs | cohort_1902 | Male | 149.7071 | 127.5074 | 175.7719 |
| DALYs | cohort_1907 | Male | 96.2289 | 88.3867 | 104.7668 |
| DALYs | cohort_1912 | Male | 66.5297 | 62.4769 | 70.8454 |
| DALYs | cohort_1917 | Male | 43.7298 | 41.4783 | 46.1036 |
| DALYs | cohort_1922 | Male | 26.9168 | 25.6656 | 28.2289 |
| DALYs | cohort_1927 | Male | 16.9169 | 16.1831 | 17.6839 |
| DALYs | cohort_1932 | Male | 10.6031 | 10.1677 | 11.0571 |
| DALYs | cohort_1937 | Male | 6.7046 | 6.4403 | 6.9798 |
| DALYs | cohort_1942 | Male | 4.1895 | 4.0287 | 4.3567 |
| DALYs | cohort_1947 | Male | 2.5815 | 2.4851 | 2.6816 |
| DALYs | cohort_1952 | Male | 1.583 | 1.5251 | 1.6432 |
| DALYs | cohort_1957 | Male | 1 | 1 | 1 |
| DALYs | cohort_1962 | Male | 0.6336 | 0.6061 | 0.6623 |
| DALYs | cohort_1967 | Male | 0.4402 | 0.4192 | 0.4623 |
| DALYs | cohort_1972 | Male | 0.2829 | 0.2656 | 0.3014 |
| DALYs | cohort_1977 | Male | 0.1901 | 0.1742 | 0.2074 |
| DALYs | cohort_1982 | Male | 0.1271 | 0.113 | 0.1431 |
| DALYs | cohort_1987 | Male | 0.0821 | 0.0709 | 0.095 |
| DALYs | cohort_1992 | Male | 0.0408 | 0.0339 | 0.049 |
| DALYs | cohort_1997 | Male | 0.0221 | 0.0183 | 0.0266 |
| DALYs | cohort_2002 | Male | 0.0104 | 0.0086 | 0.0126 |
| DALYs | cohort_2007 | Male | 0.0051 | 0.0042 | 0.0062 |
| DALYs | cohort_2012 | Male | 0.0027 | 0.0022 | 0.0033 |
| DALYs | cohort_2017 | Male | 0.0012 | 0.0009 | 0.0015 |
